# Collectin-11 promotes cancer cell proliferation and tumor growth

**DOI:** 10.1172/jci.insight.159452

**Published:** 2023-03-08

**Authors:** Jia-Xing Wang, Bo Cao, Ning Ma, Kun-Yi Wu, Wan-Bing Chen, Weiju Wu, Xia Dong, Cheng-Fei Liu, Ya-Feng Gao, Teng-Yue Diao, Xiao-Yun Min, Qing Yong, Zong-Fang Li, Wuding Zhou, Ke Li

**Affiliations:** 1 Core Research Laboratory, The Second Affiliated Hospital, Xi’an Jiaotong University, Xi’an, China.; 2 Peter Gorer Department of Immunobiology, School of Immunology & Microbial Sciences, Faculty of Life Sciences & Medicine, King’s College London, London, United Kingdom.; 3 Department of Ophthalmology, The First Affiliated Hospital, Sun Yat-sen University, Guangdong, China.; 4 Key Laboratory of Shaanxi Province for Craniofacial Precision Medicine Research, College of Stomatology, Xi’an Jiaotong University, Xi’an, China.

**Keywords:** Immunology, Oncology, Cancer, Macrophages, Melanoma

## Abstract

Collectin-11 (CL-11) is a recently described soluble C-type lectin that has distinct roles in embryonic development, host defence, autoimmunity, and fibrosis. Here we report that CL-11 also plays an important role in cancer cell proliferation and tumor growth. Melanoma growth was found to be suppressed in *Colec11*^–/–^ mice in a s.c. B16 melanoma model. Cellular and molecular analyses revealed that CL-11 is essential for melanoma cell proliferation, angiogenesis, establishment of more immunosuppressive tumor microenvironment, and the reprogramming of macrophages to M2 phenotype within melanomas. In vitro analysis revealed that CL-11 can activate tyrosine kinase receptors (EGFR, HER3) and ERK, JNK, and AKT signaling pathways and has a direct stimulatory effect on murine melanoma cell proliferation. Furthermore, blockade of CL-11 (treatment with L-fucose) inhibited melanoma growth in mice. Analysis of open data sets revealed that *COLEC11* gene expression is upregulated in human melanomas and that high *COLEC11* expression has a trend toward poor survival. CL-11 also had direct stimulatory effects on human tumor cell proliferation in melanoma and several other types of cancer cells in vitro. Overall, our findings provide the first evidence to our knowledge that CL-11 is a key tumor growth–promoting protein and a promising therapeutic target in tumor growth.

## Graphical abstract

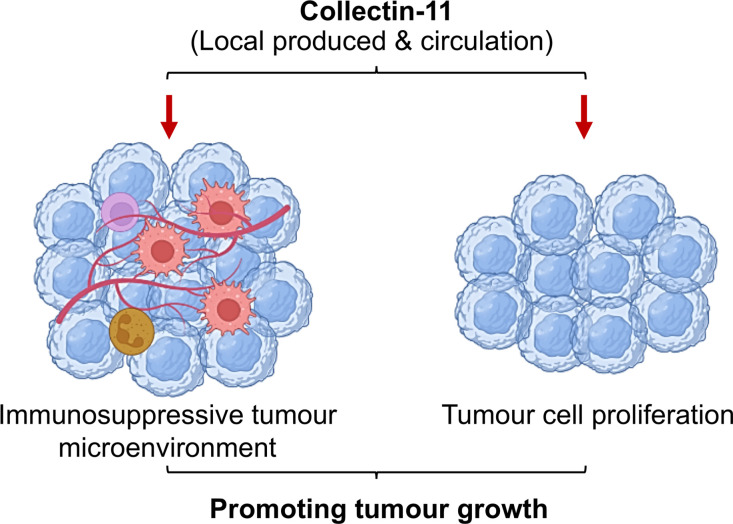

## Introduction

Collectins are group of soluble C-type lectins that represent an important group of pattern-recognition molecules. Collectins are composed of a carbohydrate-recognition domain, a neck region, and a collagen-like region, and they are involved in carbohydrate recognition and innate immunity (1, 2). Collectins can bind to carbohydrate structures on pathogens to enhance phagocytosis of pathogen by phagocytes. Some of collectins can also trigger the lectin pathway of complement activation and generate complement effector functions (3, 4). Mannose-binding lectin (MBL) and lung surfactant proteins (SP-A, SP-D) are well known members among the group.

Collectin-11 (CL-11, also known as CL-K1 and encoded by *COLEC11*) is a recently described member of the collectin family. CL-11 displays structural similarities with other collectins but has some distinct features such as having a wide tissue distribution, relatively lower serum concentrations (~300 ng/mL), and the ability to bind a wide range of ligands (5–8). These features lead to suggestions that CL-11 could be involved in a wide range and different types of cellular processes through local action, and CL-11 produced by different tissues/cells can participate in those cellular processes. CL-11 is highly conserved among species; human and mice are 92% homologous at the amino acid level (6). Carbohydrate specificity studies have shown that CL-11 preferentially binds to fucose, mannose, and high-mannose oligosaccharides present on both self- and non–self-structures (6, 8, 9). CL-11 has been shown to play important roles in embryonic development, host defence, debris removal, and regulation of cytokine secretion (10–12). CL-11 has also been shown to contribute to tissue injury and fibrosis in sterile inflammation (13, 14). More recently, in a mouse model of collagen-induced rheumatoid arthritis, we have shown that *Colec11^–/–^* mice developed more severe arthritis than WT littermates. Disease severity was associated with significantly enhanced antigen-presenting cell (APC) activation, Th1/Th17 responses, and pathogenic IgG2a production, as well as elevated circulating levels of inflammatory cytokines, indicating an important role for CL-11 in regulating APC activation and limiting autoimmunity (15). Taken together, these findings suggest that CL-11 is a multifunctional molecule, not only playing roles in homeostasis and host defence, but also participating in the pathogenesis of inflammatory and immunological diseases. So far, it is unknown whether CL-11 plays an important role in tumor growth.

Melanoma is a malignant tumor that arises from uncontrolled proliferation of melanocyte pigment–producing cells (16). Melanoma is a potentially lethal form of cancer that accounts for the majority of skin cancer deaths (17). The worldwide incidence of melanoma has steadily increased over the last several decades (18). Global cases of melanoma skin cancer will reach nearly half a million by 2040, an increase of 62% on 2018 figures (19). In the past decade, new immunotherapies, together with early diagnosis and better prevention, significantly improved cancer treatment. Immune checkpoint blockade (ICB) with monoclonal antibodies directed at the inhibitory immune receptors CTLA-4, PD-1, and PD-L1 has emerged as a successful treatment approach for patients with melanoma and other cancers (20, 21). However, these immunotherapies have only shown significant benefit for a minority of patients; the general toxicity- and immune-related adverse effects seen in the majority of patients who receive the combination therapies significantly limit their clinical use (22). Therefore, better understanding of the immunological mechanisms involved in cancer cell proliferation and tumor growth, and identifying novel tumor growth promoting molecules, are a still unmet need.

Given the role of CL-11 in stimulating fibroblast proliferation, immune regulation, and suppressing autoimmunity (12, 14, 15), we hypothesized that CL-11 may play important roles in tumor cell proliferation and tumor growth. Mouse melanoma models have been widely used for studying tumor growth, regulating tumor microenvironment (TME) and immune responses to tumors, and evaluating the efficacy in cancer therapies (23). In the present study, we employed a murine s.c. B16 melanoma model (24) combining *Colec11^–/–^* mice (25) and blockade of CL-11 with its preferential ligand approaches to determine the roles of CL-11 in melanoma cell proliferation and tumor growth. The effect of CL-11 in tumor growth was also assessed in a more clinically relevant model using YUMM1.7 murine melanoma cell line (26). We also investigated the underlying mechanisms by which CL-11 promotes tumor growth. Specifically, we explored the direct effect of CL-11 on melanoma cell proliferation and the involved receptors/signal transduction pathways and investigated the influence of CL-11 on immunosuppressive TME. Furthermore, we evaluated the relevance of CL-11 in human melanomas by analyzing *COLEC11* gene expression in human melanomas and its association with patient survival using several open data sets and assessing whether CL-11 has stimulatory effects on human melanoma cells in vitro. Moreover, we assessed the generality of CL-11 in cancer cell proliferation by examining the effects of CL-11 on cell proliferation in several human cancer cell lines in vitro.

## Results

### Melanoma growth is suppressed in Colec11^–/–^ mice.

**Figure 1:**
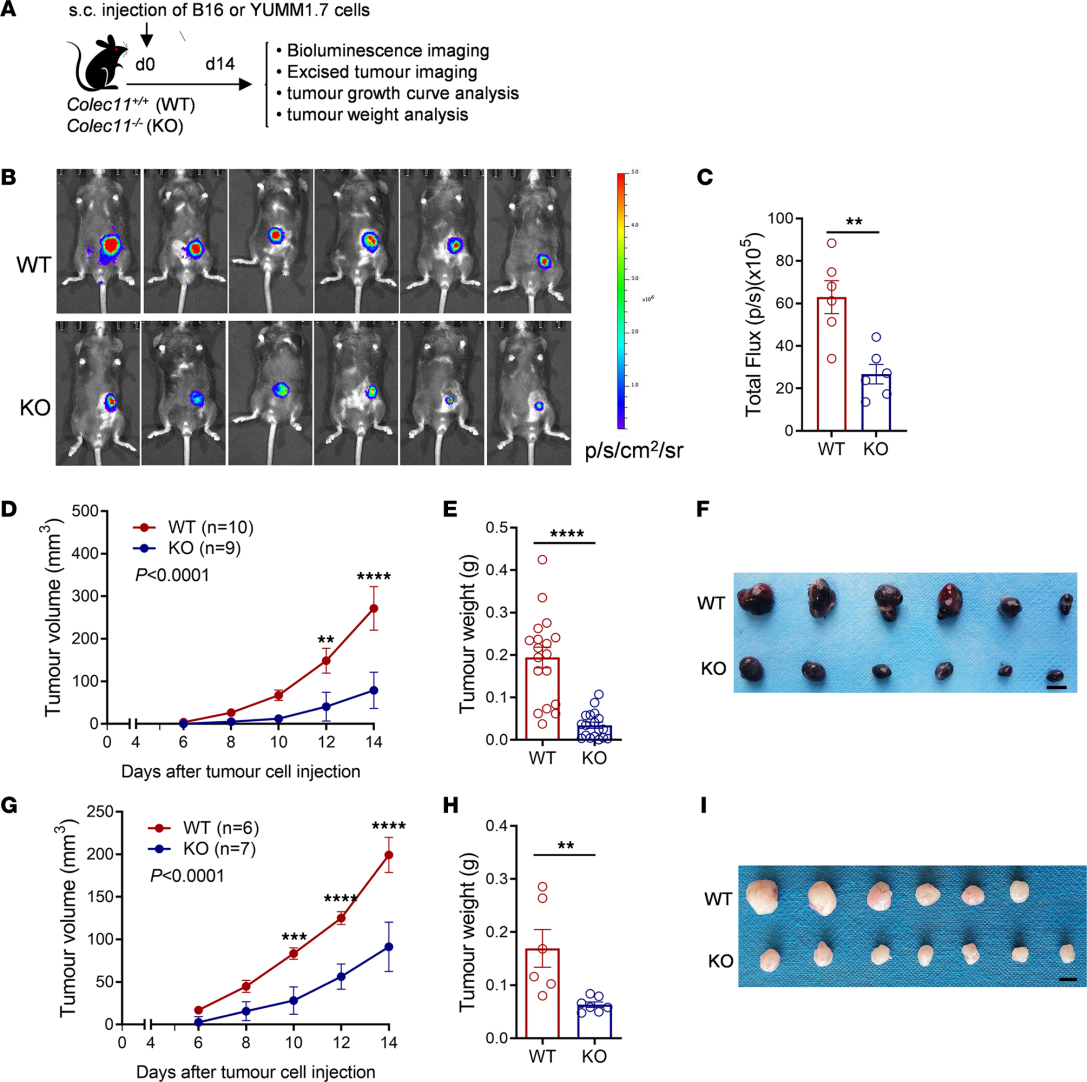
Melanoma growth is suppressed in *Colec11*^–/–^ mice. (**A**) Schematic diagram of experimental design. *Colec11^–/–^* (WT) or *Colec11^–/–^* (KO) mice received melanoma cells (luciferase-labelled B16 [B16-luc] or unlabeled B16 or YUMM1.7) by s.c. injection. (**B**) Bioluminescence images of s.c. tumors in the 2 groups of mice 14 days after injection of B16-luc cells. (**C**) Quantification of total flux (photons per second [p/s]) (the bioluminescent signal is expressed in p/s/cm2/sr in the mice shown in **B**; data are analyzed by unpaired *t* test with Welch’s correction (*n* = 6 mice per group). (**D**–**F**) B16 tumor growth. (**D**) Tumor volume (d6–d14). Data were analyzed by 2-way ANOVA with multiple-comparison test. Each symbol represents the mean of a group of mice (*n* = 9 or 10 mice/group, pooled from 2 experiments). (**E**) Tumor weight (d14). Data were analyzed by unpaired *t* test (*n* = 18 mice/group, pooled from 4 experiments). Each dot represents an individual mouse. (**F**) Images of excised tumors from the 2 groups of mice. Scale bar: 5 mm. Representative images from 2 independent experiments are shown. (**G**–**I**) YUMM1.7 tumor growth. (**G**) Tumor volume (d6–d14). Data were analyzed by 2-way ANOVA with multiple-comparison test. Each symbol represents the mean of a group of mice (*n* = 6 or 7 mice/group). (**H**) Tumor weight (d14). Data were analyzed by unpaired *t* test (*n* = 6 or 7 mice/group). Each dot represents an individual mouse. (**I**) Images of excised tumors from the 2 groups of mice. Scale bar: 5 mm. **P < 0.01; ***P < 0.001; ****P < 0.0001.

To determine the role of CL-11 in tumor growth, we assessed tumor growth in *Colec11^–/–^* mice and their WT littermates after s.c. inoculation with B16 melanoma cells by performing bioluminescence imaging and measuring tumor volume and weight (Figure 1A). Bioluminescence imaging was performed in living animals 14 days after the inoculation of luciferase-labeled melanoma cells. *Colec11^–/–^* mice had significantly reduced tumor burden compared with WT mice (Figure 1, B and C). Tumor volume changes was measured in living animals daily from day 6 (d6) to d14 after the inoculation; tumor weight was measured in excised melanomas at the end time point (d14). The tumor volume and weight were significantly reduced in *Colec11^–/–^* mice compared with WT mice (Figure 1, D–F). Similar results were observed when the YUMM1.7 melanoma model was used (Figure 1, G–I). These results demonstrate that melanoma growth is suppressed in *Colec11^–/–^* mice, indicating a role for CL-11 in promoting tumor growth.

### Colec11^–/–^ mice have reduced melanoma cell proliferation and angiogenesis.

**Figure 2:**
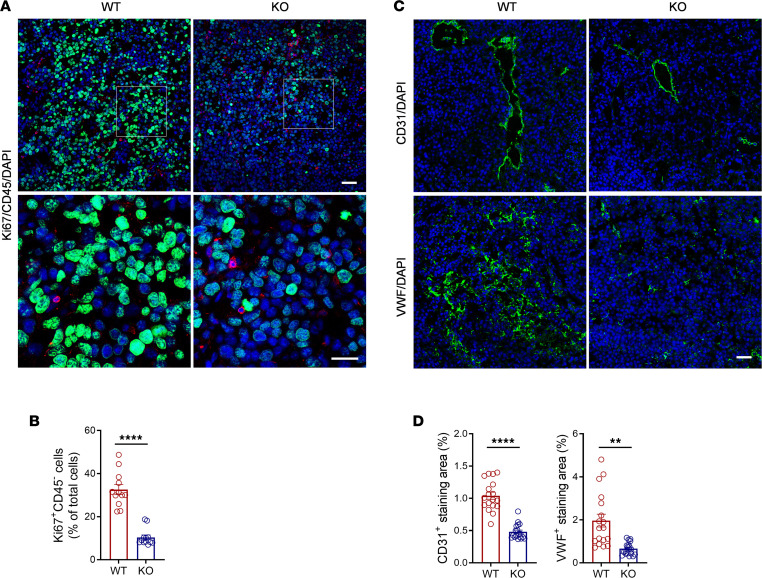
*Colec11^–/–^* mice have reduced melanoma cell proliferation and angiogenesis. Tumors excised from *Colec11^+/+^* (WT) or *Colec11^–/–^* (KO) mice (d14) were used for analysis of tumor cell proliferation, angiogenesis, and CL-11 expression. (**A**) Representative microscopy images of immunochemical staining for Ki67 (green)/CD45 (red)/DAPI (blue) in tumor core area. The bottom panel show higher-magnification images of boxed regions in the panel above. Scale bars: 50 μm (top panel), 25 μm (bottom panel). (**B**) Quantification of Ki67^+^/CD45^–^ cells corresponding to the 2 groups of mice in **A**. Data were analyzed by unpaired *t* test (*n* = 12; 3 mice, 4 image regions from each tumor section each mouse). (**C**) Representative microscopy images of immunochemical staining for CD31 (green)/DAPI (blue) or VWF (green)/DAPI (blue) in tumor core area. Scale bar: 50 μm. (**D**) Quantification of CD31- or VWF-stained areas corresponding to the WT and KO mice in **C**. Data were analyzed by unpaired *t* test (*n* = 18; 3 mice, 6 image regions from each tumor section each mouse). **P < 0.01; ****P < 0.0001.

Tumor cell proliferation and angiogenesis are among the hallmarks of tumor growth; we therefore examined these 2 parameters in excised melanomas (d14). Tumor cell proliferation was assessed by immunochemical staining of Ki67 and CD45 (used for identifying/excluding non-B16 cells). Compared with WT mice, *Colec11^–/–^* mice displayed a significant reduction of Ki67^+^ cells in the tumor core, most of the Ki67^+^ staining was not associated with CD45^+^ staining, confirming that Ki67^+^ cells are mainly tumor cells (Figure 2, A and B). Angiogenesis was assessed by immunochemical staining of endothelial markers (CD31, von Willebrand factor [VWF]). CD31 and VWF were markedly reduced in melanomas of *Colec11^–/–^* mice compared with WT mice. CD31 is an adhesion molecule and is expressed on endothelial cells, while VWF is stored in Weibel-Palade bodies of endothelial cells, which are released upon endothelial cell activation. Accordingly, different patterns of endothelial staining were observed, while CD31 was mainly detected on larger vessels, and VWF seemed mainly on smaller vessels (Figure 2, C and D). These results demonstrate that *Colec11^–/–^* mice have reduced melanoma cell proliferation and angiogenesis, indicating that CL-11 is required for tumor cell proliferation and angiogenesis.

### Detection of CL-11 in melanomas.

In order to understand how CL-11 influences tumor growth, we examined whether CL-11 is present within melanomas, whether CL-11 can be produced locally, and their cellular sources. We performed IHC staining of CL-11 in melanomas of WT and *Colec11^–/–^* mice to evaluate protein expression. CL-11 was abundantly detected in the melanomas of WT mice, while it was detected much less in the melanomas of *Colec11^–/–^* mice (Supplemental Figure 1A; supplemental material available online with this article; https://doi.org/10.1172/jci.insight.159452DS1). It is known that CL-11 can be produced by a wide variety of tissues/cell types, including skin, spleen, bone marrow, and lymph node (27); in the circulation, CL-11 is often complexed with another collectin (CL-10) forming high-order oligomers at sizes up to 750 kDa that may not be sufficiently extravasated (28). Therefore, it is conceivable that immune-infiltrating cells and nonimmune cells within melanomas could contribute to the local pool of CL-11. We therefore assessed local production of CL-11 and their cellular sources. Quantitative PCR (qPCR) showed that *Colec11* mRNA was detected in the melanomas of both WT and *Colec11^–/–^* mice; however, significantly higher expression was observed in WT mice, compared with *Colec11^–/–^* mice (Supplemental Figure 1B). Costaining of CL-11 and CD45 showed that, in the melanomas of WT mice, the majority of CD45^+^ cells were positively stained with CL-11, whereas in the melanomas of *Colec11^–/–^* mice, CD45^+^ cells appeared to be negative for CL-11 staining (Supplemental Figure 1C). Cellular sources of CL-11 in the melanoma were further analyzed by qPCR in CD45^+^ (immune-infiltrating) cells and CD45^–^ (nonimmune) cells obtained from melanomas of WT and *Colec11^–/–^* mice by FACS. In the cell preparations from WT mice, *Colec11* mRNA was mainly detected in CD45^+^ cells, while it was detected at very low levels in CD45^–^ cells. In the cell preparations from *Colec11^–/–^* mice, no *Colec11* mRNA was detected in CD45^+^ cells, and — just as in WT mice — only very low levels were detected in CD45^–^ cells, implying there is a small amount of CL-11 being produced by melanoma cells (Supplemental Figure 1D). Collectively, these results demonstrate that CL-11 — which can be produced locally — is present in the melanomas; CD45^+^ infiltrating leukocytes are the major cellular source of CL-11 in the melanoma; and melanoma cell can produce small amount of CL-11.

### Colec11^–/–^ mice exhibit less immunosuppressive TME.

The TME is an important regulator of tumor growth. Although, in general, the TME exhibits immunosuppressive features, it could be more or less immunosuppressive, depending on cancer-immunoediting processes that could potentially be regulated by factors in the TME. Therefore, we investigated whether CL-11 has an effect on the TME. In doing this, we performed several cellular and molecule analyses using excised B16 melanomas from WT and *Colec11^–/–^* mice to assess: (a) composition of tumor-infiltrating leukocytes, (b) intratumor gene expression of cytokines/chemokines, and (c) phenotype of tumor-associated macrophages (TAMs).

**Figure 3:**
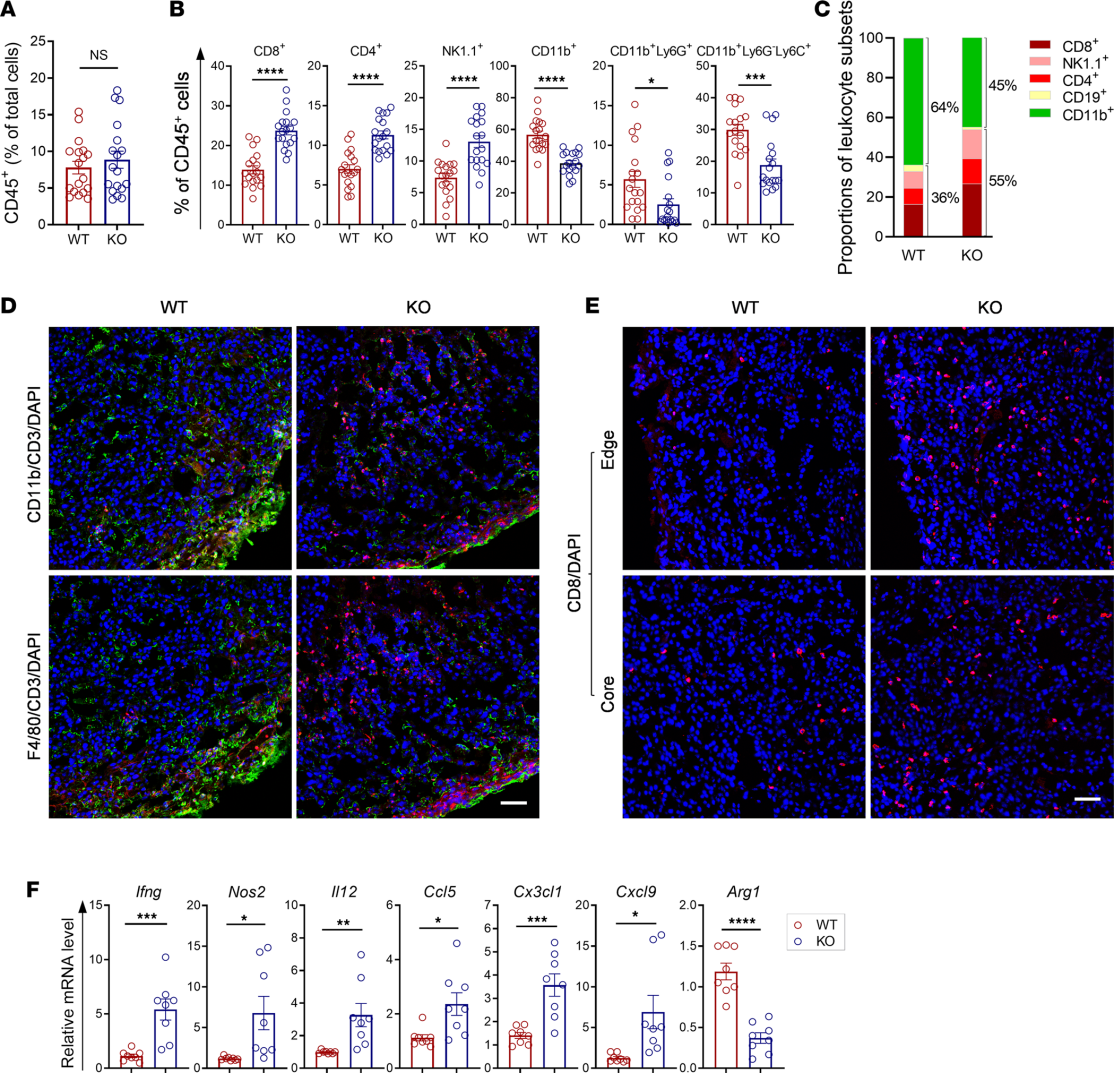
*Colec11^–/–^* mice exhibit less immunosuppressive TME. Tumors excised from *Colec11^+/+^* (WT) or *Colec11^–/–^* (KO) mice (d14) were used for analyzing TME. (**A**–**C**) Tumor infiltrates analyzed by flow cytometry. (**A**) CD45^+^ cells. (**B**) Subsets of tumor-infiltrating leukocytes analyzed by flow cytometry. Data were analyzed by unpaired *t* test (*n* = 18 mice per group, pooled from 4 experiments). Each dot represents an individual mouse. (**C**) A bar chat representing proportion of subsets in CD45^+^ cells shown in **B**. (**D**) Representative microscopy images of immunochemical staining for CD11b (green)/CD3 (red)/DAPI (blue) and F4/80 (green)/CD3 (red)/DAPI (blue). Scale bar: 50 μm. (**E**) Representative microscopy images of immunochemical staining for CD8 (red)/DAPI (blue) in tumor edge and core areas. Scale bar: 50 μm. (**F**). qPCR analysis in tumor tissues. Data were analyzed by unpaired *t* test (*n* = 8 mice per group). *P < 0.05; **P < 0.01; ***P < 0.001; ****P < 0.0001.

The composition of tumor-infiltrating leukocytes in melanomas was evaluated by flow cytometry and immunochemical staining. Flow cytometry analysis showed that the percentage of tumor-infiltrating CD45^+^ cells was comparable between WT and *Colec11^–/–^* mice (Figure 3A). However, the subsets of CD45^+^ cells were markedly different between the 2 groups of mice. Compared with WT mice, *Colec11^–/–^* mice had higher proportions of lymphocytes (CD4^+^, CD8^+^, NK1.1^+^) and lower proportions of myeloid lineage cells (CD11b^+^, CD11b^+^Ly6G^+^, CD11b^+^Ly6G^–^Ly6G^+^) (Figure 3B). A lower percentage of CD25^+^ cells within the CD4^+^ T cell compartment was detected in melanomas of *Colec11^–/–^* mice compared with WT mice (Supplemental Figure 2, A and B). There was no significant difference in CD19^+^ B cells within the CD45^+^ population between the 2 groups of mice (Supplemental Figure 2C). This led to a relatively higher lymphocytes-to-myeloid cells ratio in *Colec11^–/–^* mice than that in WT mice (Figure 3C). The costaining of CD3 and CD11b or CD3 and F4/80 showed that, compared with WT mice, *Colec11^–/–^* mice exhibited clearly more CD3^+^ infiltrates and much less CD11b^+^ and F4/80^+^ infiltrates in the tumor core and in the outskirt of the tumors (Figure 3D). Furthermore, the staining of CD8 showed that, compared with WT mice, *Colec11^–/–^* mice exhibited markedly increased CD8^+^ infiltrates in the tumor core and tumor edge (Figure 3E). More CD3^+^ infiltrates and fewer CD11b^+^ infiltrates were also observed in *Colec11^–/–^* mice when YUMM1.7 melanoma model was used (Supplemental Figure 3). Collectively, these results demonstrate that *Colec11^–/–^* mice have a higher lymphocytes/myeloid lineage cells ratio and a better cytotoxic T cell infiltration in the melanoma.

Intratumor mRNA levels of cytokines/chemokines highly relevant to the TME were analyzed by qPCR. Significantly higher levels of *Ifng*, *Nos2*, *Il12*, *Ccl5*, *Cx3cl1*, and *Cxcl9* — and, by contrast, significantly lower levels of Arg1 — were detected in the melanomas of *Colec11^–/–^* mice, compared with WT mice (Figure 3F). These results demonstrate that the production of cytokines/chemokines having antitumor properties is upregulated in the melanomas of *Colec11^–/–^* mice.

**Figure 4:**
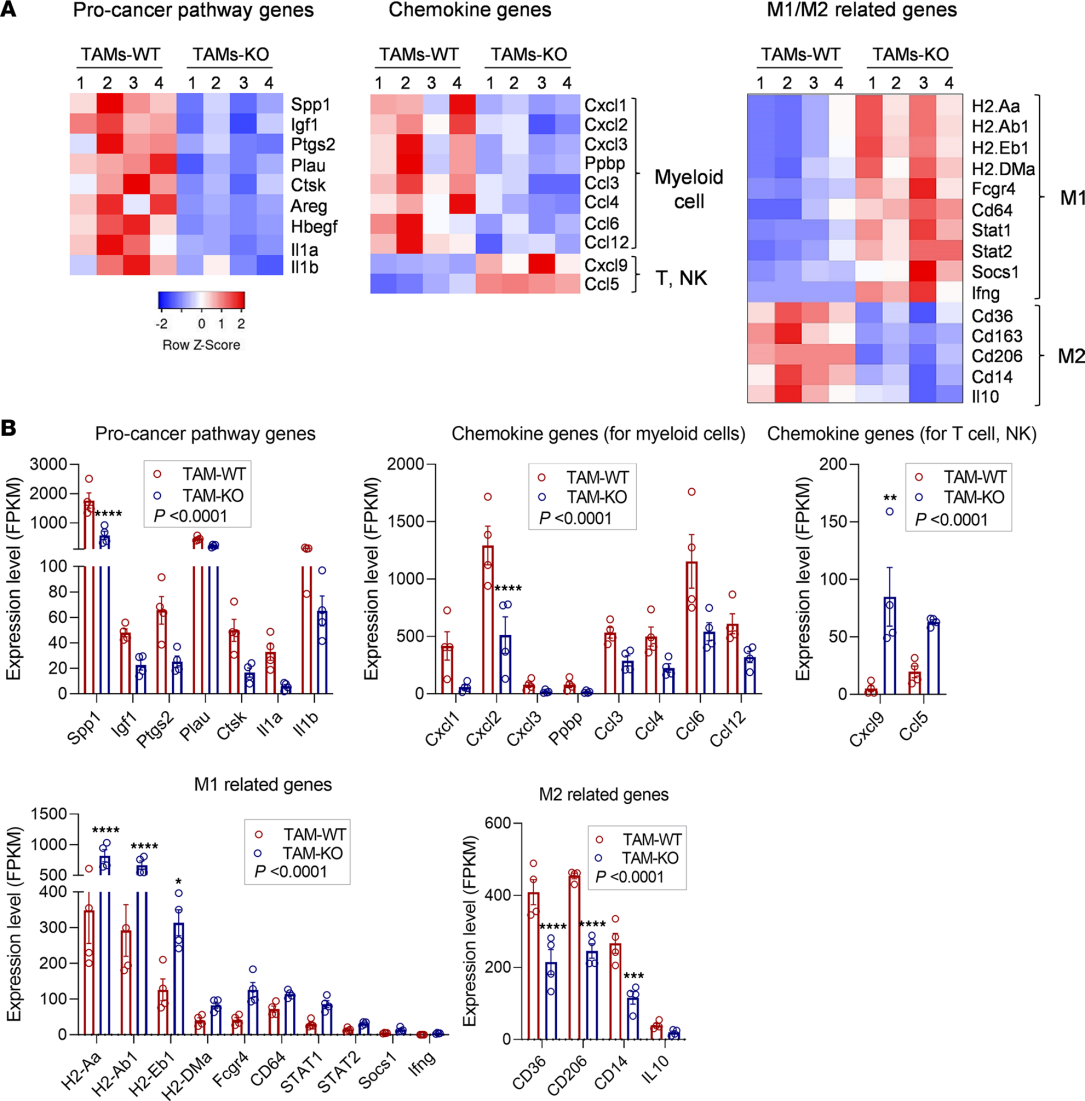
TAMs from *Colec11^–/–^* mice exhibit less immunosuppressive features. Tumors excised from *Colec11^+/+^* (WT) or *Colec11^–/–^* (KO) mice (d14) were used for analyzing TAMs phenotypes by RNA-Seq. (**A**) Heatmaps of RNA-Seq data showing genes with significant differential expression (with fold change ≥ 1.5) between TAMs (CD45^+^F4/80^+^) from WT mice (TAM-WT) and KO mice (TAM-KO). Left panel: procancer pathway genes. Middle panel: chemokine genes encoding chemokines responsible for immune cell (myeloid cell, T cell, and NK cell) recruitment. Right panel: M1/M2-related genes. (**B**) FPKM gene expression levels corresponding to the 2 groups of TAMs in **A**. Data were analyzed by 2-way ANOVA with multiple-comparison test (*n* = 4 mice/group). *P < 0.05; **P < 0.01; ***P < 0.001; ****P < 0.0001.

TAMs are considered as main components of the TME and are present in high numbers in the microenvironment of solid tumors. TAMs affect most aspects of tumor cell biology and drive pathological phenomena, including tumor cell proliferation, tumor angiogenesis, invasion, and metastasis, as well as suppression of antitumor immune responses (29). We therefore performed RNA-Seq in TAMs isolated from melanomas of WT and *Colec11^–/–^* mice by sorting CD45^+^F4/80^+^ cells by FACS. RNA-Seq analysis revealed 625 differentially expressed genes (DEGs) (log_2_FC ≥ 1). These genes are mainly classified into “Immune system,” “Signal transduction,” “Signaling molecules and interaction” and “Cancer” pathways (Supplemental Figure 4). DEGs in Immune system and Cancer pathways were further analyzed focusing on genes involved in tumor progression/invasion/metastasis and MΦ phenotype. Compared with TAMs from WT mice, TAMs from *Colec11^–/–^* mice exhibited: (a) a lower expression of genes coding for procancer molecules (*Spp1, Igf1, Plgs2, Plau, Ctsk, Areg, Hbegf, Il1a, Il1b*); (b) a lower expression of genes coding for chemoattractant of myeloid-derived suppressor cells (MDSC) and neutrophil (*Cxcl1, Cxcl2, Cxcl12, Cxcl3, Pbbp*) and MO/MF (*Ccl3, Ccl4, Ccl6, Ccl12*) and, by contrast, a higher expression of genes coding for potent chemoattractant of T cell and NK cell (Cxcl9, Ccl5); and (c) a higher expression of M1 marker genes (*H2.Aa, H2.Ab1, H2.Eb1, H2.DMa, Fcgg4, CD64, Stat1, Stat2, Socs1, Ifng*) and, by contrast, a lower expression of M2 marker genes (*Cd36, Cd163, Cd206, Cd14, Il10*) (Figure 4, A and B). These results demonstrate that TAMs from *Colec11^–/–^* mice exhibit less immunosuppressive and tumor-suppressing features, while TAMs from WT mice exhibit more immunosuppressive and tumor-promoting features.

**Figure 5:**
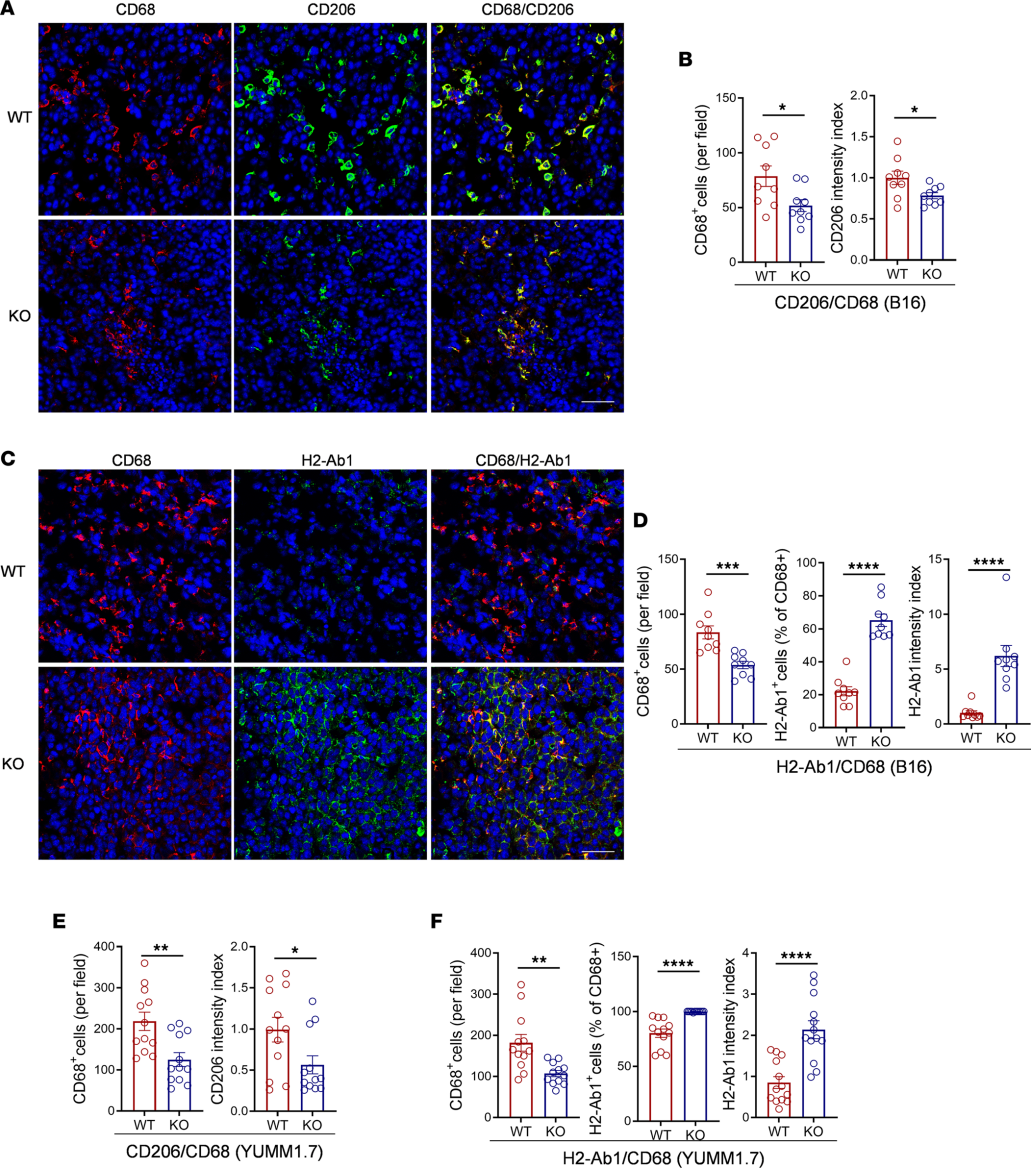
Effect of CL-11 deficiency on CD206 and MHC class II expression in TAMs. B16 or YUMM1.7 tumors excised from *Colec11^+/+^* (WT) or *Colec11^–/–^* (KO) mice (d14) were used for analyzing TAM phenotypes by IHC. (**A**–**F**) B16 Tumors. (**A**) Representative microscopy images of costaining for CD68 (red), CD206 (green), and DAPI (blue). Scale bars: 50 μm. (**B**) Quantification of total CD68^+^ cells (data are expressed as number of CD68^+^ cells per field [0.34 mm^2^]) and CD206 fluorescence intensity in CD68^+^ cells (data are presented as normalized intensity index). (**C**) Representative microscopy images of costaining for CD68 (red), H2-Ab1 (green), and DAPI (blue). Scale bars: 50 μm. (**D**) Quantification of total CD68^+^ cells, percentage of H2-Ab1^+^ cells in total CD68^+^ cells, and H2-Ab1 fluorescence intensity in CD68^+^ cells. (**B** and **D**) Data were analyzed by unpaired *t* test (*n* = 9; 3 mice, 3 image regions from each tumor section each mouse). (**E** and **F**) YUMM1.7 tumors. (**E**) Quantification of total CD68^+^ cells and CD206 fluorescence intensity in CD68^+^ cells. (**F**) Quantification of total CD68^+^ cells, percentage of H2-Ab1^+^ cells in total CD68^+^ cells, and H2-Ab1 fluorescence intensity in CD68^+^ cells. (**E** and **F**) Data were analyzed by unpaired *t* test (*n* = 12; 3 mice, 4 image regions from each tumor section each mouse). *P < 0.05; **P < 0.01; ****P < 0.0001.

M1/M2 phenotypes of TAMs in the melanomas of WT and *Colec11^–/–^* mice were further evaluated by IHC costaining of CD206/CD68 or H2-Ab1/CD68. Costaining analysis of CD206/CD68 showed that the melanomas of *Colec11^–/–^* mice exhibited lower numbers of CD68^+^ cells than WT controls. Although the percentage of CD206^+^ cells in CD68^+^ cells had no difference between the 2 groups (as almost all of CD68^+^ cells in both WT and *Colec11^–/–^* mice were positive for CD206), the fluorescence intensity of CD206 in CD68^+^ cells was lower in the melanomas of *Colec11^–/–^* mice than WT controls (Figure 5, A and B). Costaining analysis of MHC class II (H2-Ab1)/CD68 showed that the melanomas of *Colec11^–/–^* mice exhibited lower numbers of CD68^+^ cells again but higher percentage of H2-Ab1^+^ cells and higher intensity of H2-Ab1 in CD68^+^ cells than WT controls (Figure 5, C and D). These results are consistent with gene expression data in RNA-Seq analysis indicating that CL-11 deficiency leads to reprogramming TAMs to M1-like macrophages. Similar results were observed when the YUMM1.7 melanoma model was used (Figure 5, E and F).

Collectively, the above results demonstrate that the TME in *Colec11^–/–^* mice is less immunosuppressive, indicating that CL-11 is required for establishment of more immunosuppressive TME.

### CL-11 induces mitogenic kinase signaling and stimulates melanoma cell proliferation in vitro.

**Figure 6:**
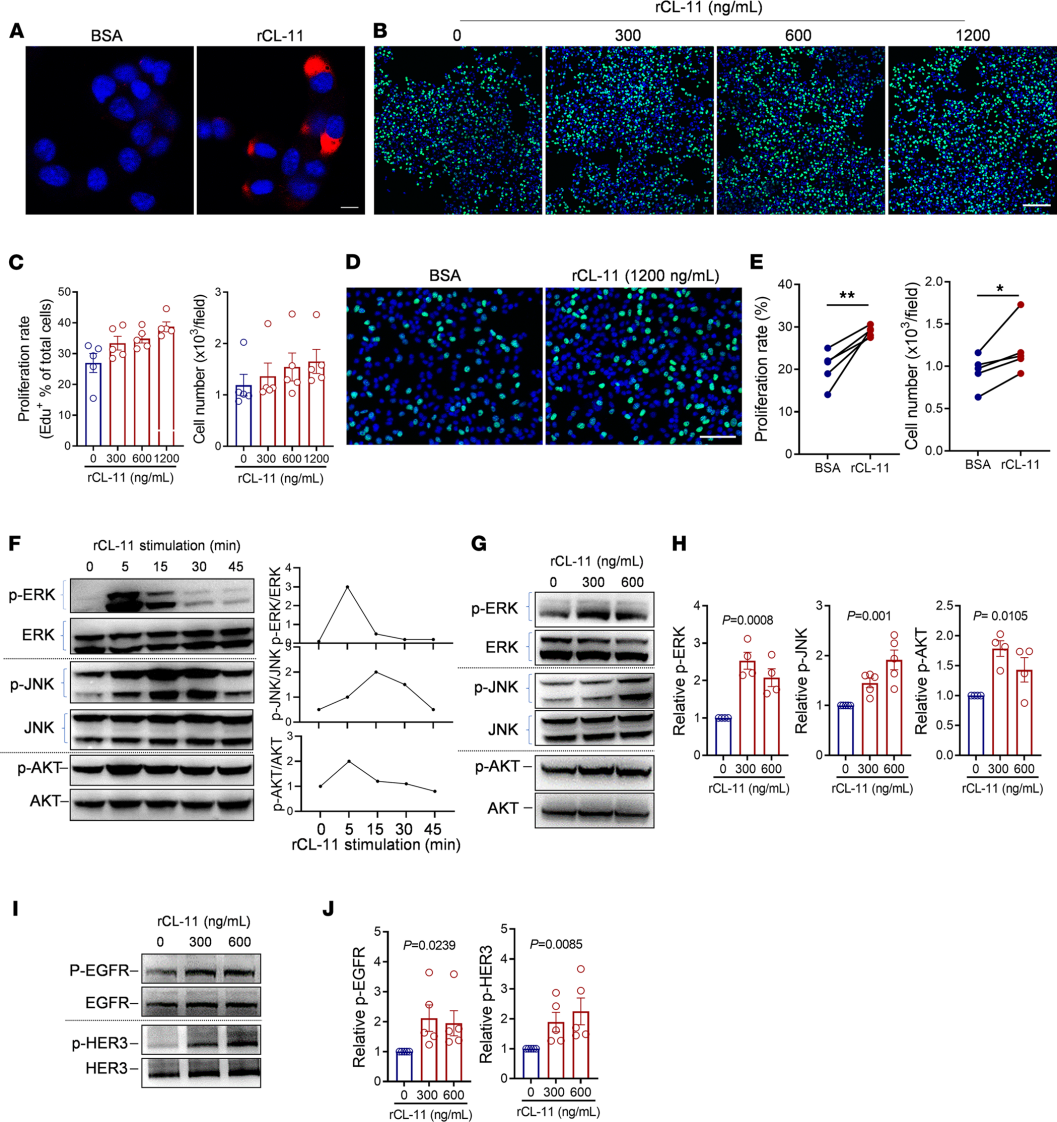
CL-11 induces mitogenic kinase signaling and stimulates melanoma cell proliferation in vitro. (**A**) Representative microscopy images showing CL-11 bound to B16 cells. CL-11 (red) and DAPI (blue). Scale bar: 10 μm. (**B**) Representative microscopy images of EdU staining in B16 cells following the treatment with rCL-11 at the indicated concentrations. EdU (green) and DAPI (blue). Scale bar: 200 μm. (**C**) Cell proliferation rate and total cell numbers corresponding to the images in **B**. **B** and **C** are representative of 2 independent experiments. Data are expressed as mean ± SEM. (**D** and **E**) Additional sets of EdU cell proliferation assay performed using 1,200 ng/mL of rCL-11. (**D**) Representative microscopy images of EdU staining. Scale bar: 100 μm. (**E**) Cell proliferation rate and total cell numbers. Data were analyzed by paired *t* test (5 individual experiments). Each dot represents the average value of the percentages or cell numbers calculated from 5 image fields at 100***×*** for each sample. (**F**) Left panel: Western blots showing total and phosphorylated ERK (p-ERK), JNK (p-JNK), and AKT (p-AKT) in B16 melanoma cells at the indicated time points following rCL-11 (600 ng/mL) stimulation. Right panel: quantification of phosphorylated proteins relative to their respective total proteins corresponding with the blots. (**G**) Representative Western blots showing total and p-ERK, p-JNK, and p-AKT in B16 melanoma cells that have been stimulated with rCL-11 at the indicated concentrations for 5 minutes. (**H**) Quantification of p-ERK, p-JNK, and p-AKT relative to their respective total proteins corresponding to the blots in **G**. (**I**) Representative Western blots showing total, p-EGFR, and p-HER3 in B16 cells following the treatment with rCL-11 for 5 minutes. (**J**) Quantification of p-EGFR and p-HER3 relative to their respective total proteins corresponding with the blots in **I**. (**H** and **J**) Data were analyzed by 1-way ANOVA (5 independent experiments). *P < 0.05; **P < 0.01.

Having demonstrated that CL-11 is required for melanoma cell proliferation and tumor growth in vivo, next we investigated whether CL-11 has a direct stimulatory effect in melanoma cell proliferation in vitro. We first confirmed the binding of CL-11 to B16 melanoma cells by immunochemical staining (Figure 6A). We then performed EdU proliferation assay in B16 melanoma cells following the treatment with murine recombinant CL-11 (rCL-11). Initial-dose titration experiments showed that addition of rCL-11 (at different concentrations, 300–1,200 ng/mL) into melanoma cell cultures clearly increased cell proliferation rate and total cell numbers, with a maximal increase at 1,200 ng/mL (Figure 6, B and C). The stimulatory effect of CL-11 on melanoma cell proliferation was further confirmed by repeating the assay with a concentration of rCL-11 at 1,200 ng/mL (Figure 6, D and E). These results clearly demonstrate that CL-11 has a direct stimulatory effect on melanoma cell proliferation.

Activation of mitogen-activated protein kinase (MAPK) and PI3K/AKT signaling pathways play essential roles in cell proliferation. We therefore sought to investigate whether CL-11 has effects on the activation of these signaling pathways in melanoma cells. Western blotting (WB) analysis showed that treatment of B16 cells with rCL-11 (600 ng/mL) clearly increased phosphorylation of ERK, JNK, and AKT, with peak at 5 minutes, 15 minutes, and 5 minutes, respectively (Figure 6, F and G). The stimulatory effects of CL-11 on this signaling was further confirmed by repeating the experiment at optimal time points with 2 doses of rCL-11 (300 and 600 ng/mL). Results showed that rCL-11 significantly enhanced phosphorylation of ERK, JNK, and AKT (Figure 6, H and I).

The ErbB family (also known as EGFR family) of receptor tyrosine kinase is a family of 4 receptors, including epidermal growth factor receptor (EGFR, also known as ErbB-1; HER1), ErbB-2 (HER2), ErbB-3 (HER3), and ErbB-4 (HER4). Once engaged by their ligands (mainly growth factors, certain cytokines), the receptors undergo dimerization that induces its autophosphorylation, which mediates the activation of multiple downstream signaling pathways, including MAPK and PI3K/AKT, leading to cell proliferation. We therefore sought to investigate whether CL-11–mediated melanoma cell proliferation is involved in ErbB receptors. Although the expression of ErbB receptors in many types of solid tumors including melanoma has been well documented (30, 31), inconsistent findings have been reported about the expression of these receptors in cultured melanoma cells (32, 33). We therefore first examined the expression of the 4 ErbB receptors in B16 melanoma cells. EGFR, HER2, and HER3 — but not HER4 — were clearly detected by RT-PCR and WB, confirming the expression of EGFR, HER2, and HER3 receptors in B16 melanoma cells (Supplemental Figure 5, A and B). Next, we assessed whether CL-11 can activate these receptors in melanoma cells. WB analysis showed that rCL-11 induced enhancement of phosphorylation of EGFR and HER3 in B16 cells (Figure 6J) but had no effect on HER2 phosphorylation (data not shown). 

Collectively, the above results demonstrate that CL-11 can activate EGFR, HER3 and ERK, and JNK and AKT signaling and has a direct stimulatory effect on melanoma cell proliferation.

### Treatment of mice with L-fucose inhibits melanoma growth.

**Figure 7:**
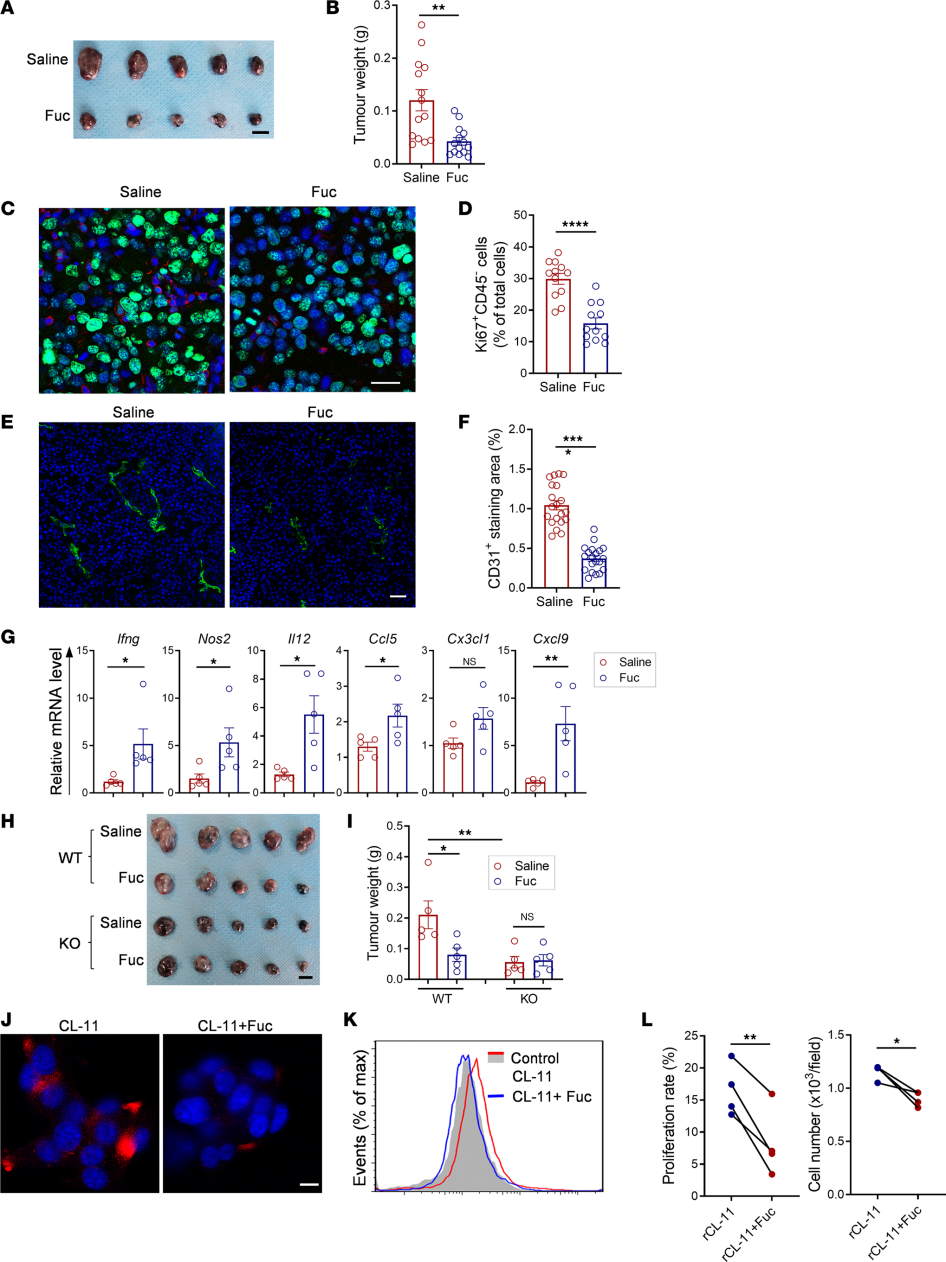
Treatment of mice with L-fucose inhibits melanoma growth. (**A**–**G**) WT mice that received treatment with saline or L-fucose (Fuc) daily by i.p. tumors were excised at d14 and used for analysis. (**A**) Representative images of tumors (*n* = 3 independent experiments). Scale bar: 5 μm. (**B**) Tumor weights. Data were analyzed by unpaired *t* test (*n* = 14 mice/group, pooled from 3 experiments). (**C**) Representative microscopy images of immunochemical staining for Ki67 (green)/CD45 (red)/DAPI (blue) in tumor core area. Scale bar: 25 μm. (**D**) Quantification of Ki67^+^/CD45^–^ cells. Data are analyzed by unpaired *t* test (*n* = 12; 4 mice, 3 fields from each tumor section). (**E**) Representative microscopy images of immunochemical staining for CD31 (red)/DAPI (blue) in tumor core area. Scale bar: 50 μm. (**F**) Quantification of CD31. Data were analyzed by unpaired *t* test (*n* = 20; 4 mice, 5 fields from each tumor). (**G**) qPCR analysis of intratumor cytokines/chemokines. Data were analyzed by unpaired *t* test (*n* = 5 mice/group). (**H** and **I**) Tumors were excised at d14 from *Colec11^+/+^* (WT) or *Colec11^–/–^* (KO) mice that received treatment with saline or Fuc daily. (**H**) Images of tumors. Scale bar: 5 μm. (**I**) Tumor weights. Data were analyzed by 1-way ANOVA with Tukey’s multiple-comparison test (*n* = 5 mice/group). **H** and **I** are representative of 2 independent experiments. (**J** and **K**) Representative microscopy images and flow cytometry histogram showing CL-11 (red) binding to B16 cells (DAPI) was blocked by Fuc (*n* = 3). Scale bar: 10 μm. (**L**) EdU proliferation assay in B16 melanoma cells following the treatment with rCL-11 or rCL-11 plus Fuc for 72 hours. Cell proliferation rate and total cell numbers were shown; each dot represents the average value of percentage of EdU^+^ cells, or cell numbers calculated from 5 image fields at 100***×*** for each sample. Data were analyzed by paired *t* test (4 independent experiments). *P < 0.05; **P < 0.01; ***P < 0.001; ****P < 0.0001.

Having demonstrated the role of CL-11 in promoting melanoma growth by using *Colec11^–/–^* mice, we next sought to explore therapeutic potential of targeting CL-11 in melanoma growth. Previous studies in the murine model of renal IR injury have shown that treatment of mice with a soluble ligand (L-fucose) for CL-11 to block CL-11 binding to the target ligand on renal ischemic tissue could inhibit CL-11–dependent renal IR injury (34). We therefore assessed whether treatment of mice with L-fucose could inhibit melanoma growth. The first set of experiments was performed in WT mice. The L-fucose or control (saline) was administered daily by i.p. injection (starting 24 hours after the inoculation of B16 melanoma cells). Tumors were excised from the mice on day 14 after the inoculation and subjected to analysis of tumor size and weight, tumor cell proliferation, angiogenesis, and gene expression of cytokines/chemokines as described in Figures 1 and 2. Compared with the control group, mice receiving treatment with L-fucose had slowed tumor growth, which was associated with reduced number of Ki67^+^ tumor cells and expression of CD31 and VWF (Figure 7, A–F) as well as elevated intratumor mRNA levels of several cytokines/chemokines having antitumor properties (*Ifng, iNos, Il12, Ccl5, Cx3cl1, Cxcl9*) (Figure 7G). The second set of experiments was performed in WT and *Colec11^–/–^* mice; the effect of L-Fucose treatment on tumor growth was assessed and compared in the 2 groups of mice. As expected, tumor size and weight were significantly reduced in WT mice receiving treatment with L-fucose, compared with the control group. However, the reduction of tumor size and weight was not observed in *Colec11^–/–^* mice receiving treatment with L-fucose compared with the control group (Figure 7, H and I). Together, these results demonstrate that treatment of mice with L-fucose suppresses melanoma growth and the suppression is dependent on the presence of CL-11.

We also assessed whether L-fucose could inhibit CL-11–mediated melanoma cell proliferation in vitro. We first confirmed that L-fucose is able to block the binding of CL-11 to B16 melanoma cells by immunocytochemistry and flow cytometry (Figure 7, J and K). Next, we performed an EdU proliferation assay in B16 melanoma cells, which were treated with rCL-11 in the presence or absence of L-fucose for 72 hours. Proliferation rate and total cell numbers were significantly reduced by the presence of L-fucose compared with the absence of L-fucose (Figure 7L). These results demonstrate that L-fucose has an inhibitory effect on CL-11–mediated melanoma cell proliferation.

### Relevance of CL-11 to human cancer.

**Figure 8:**
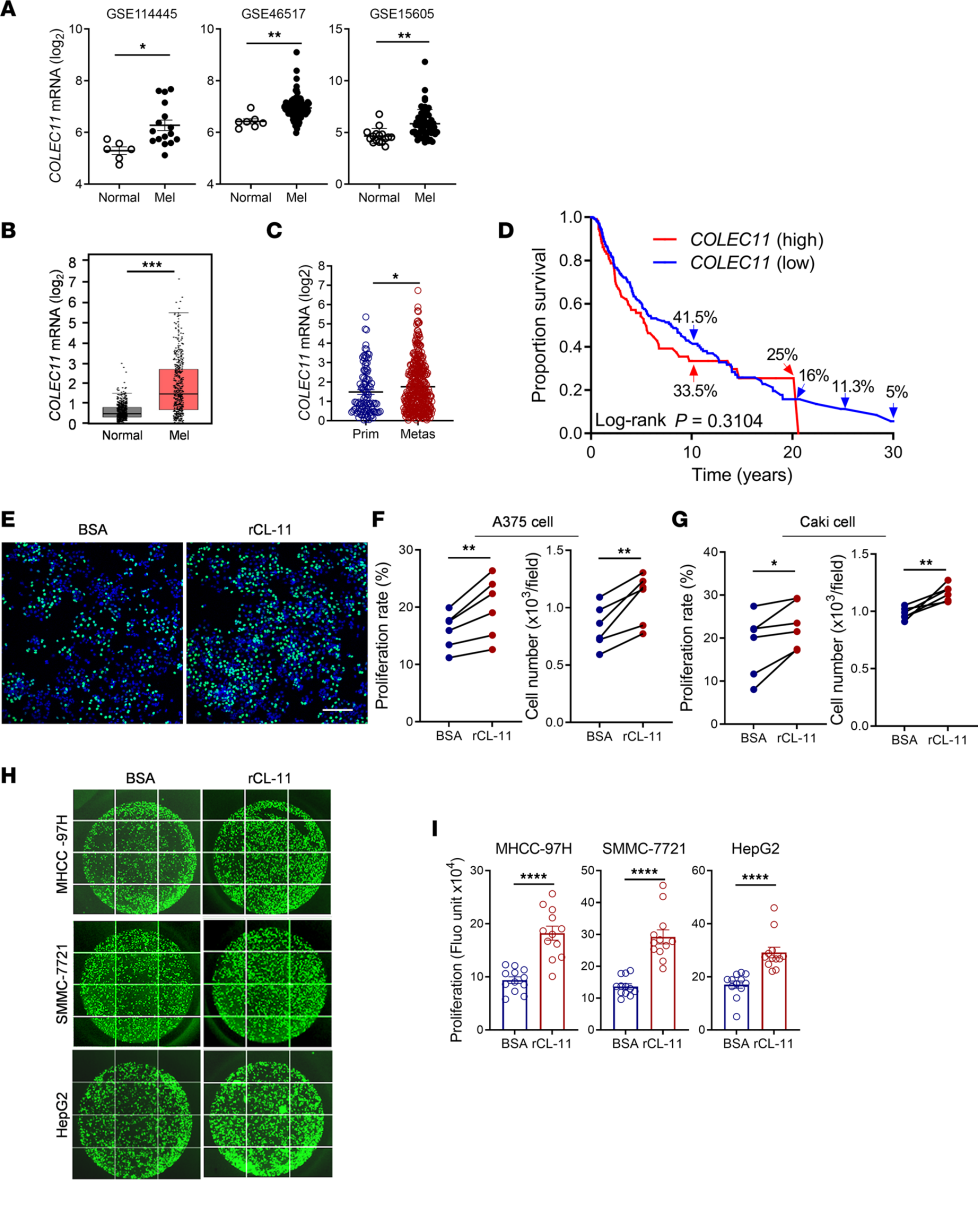
Relevance of CL-11 to human cancer. (**A** and **B**) *COLEC11* gene expression in normal human skin tissues and melanomas. (**A**) Analysis of the data from NCBI (GEO GSE114445 [normal (*n* = 6) and melanoma (*n* = 16)], GSE46517 [normal (*n* = 7), melanoma (*n* = 104)], and GSE15605 [normal (*n* = 16), melanoma (*n* = 58)]). (**B**) Analysis of the data from TCGA SKCM and GTEx (via GEPIA [normal (*n* = 588), melanomas (*n* = 471)]; ref. 38). (**A** and **B**) Data were analyzed by unpaired *t* test. (**C**) Analysis of the data obtained from TCGA SKCM (primary [*n* = 103], metastatic [*n* = 368]). Data were analyzed by Mann-Whitney *U* test. (**D**) Association of *COLEC11* overexpression with survival in patients with melanoma. Kaplan-Mayer survival curves was generated using the data obtained from TCGA SKCM. *COLEC11* expression was compared between 75% quartile (low, *n* = 344) and 25% quartile (high, *n* = 114). The survival rates at specific time points were indicated. Overall survival was analyzed using stratified multivariate log-rank test. (**E**) Representative microscopy images of EdU staining in A375 cells following the treatment with rCL-11, EdU (green) and DAPI (blue). Scale bar: 200 μm. (**F** and **G**) Quantification of cell proliferation rate and total cell numbers in A375 cells and Caki cells corresponding to the images of EdU staining. Data were analyzed by paired *t* test (6 individual experiments). (**H**) Representative images of EdU staining in 3 types of liver cancer cells (MHCC-97H, SMMC-7721, HepG2). (**I**) Quantification of cell proliferation in MHCC-97H, SMMC-7721, and HepG2 cells by measuring fluorescence intensity of EdU staining. Data were analyzed by unpaired *t* test (*n* = 12/group, pooled from 4 individual experiments). Normal, normal skins; Mel, melanomas; Prim, primary melanomas; Metas, metastatic melanomas; Fluo unit, fluorescence unit. *P < 0.05; **P < 0.01; ***P < 0.001; ****P < 0.0001.

To evaluate the relevance of CL-11 in human cancer, we analyzed *COLEC11* gene expression in human melanomas and normal skin using 4 data sets obtained from online resources (GSE114445, GSE46517, GSE15605, TCGA SKCM, and GTEx; refs. 35–38). *COLEC11* gene expression levels were significantly elevated in melanomas compared with normal skin in all 4 data sets (Figure 8, A and B). We also analyzed *COLEC11* gene expression in primary and metastatic melanomas and found higher levels in metastatic melanomas (Figure 8C). Furthermore, we evaluated the correlation of *COLEC11* expression with survival of patients with melanoma using the data set obtained from TCGA SKCM. We categorized expression levels of *COLEC11* as high (top 25% quartile, *n* = 114) and low (low 75% quartile, *n* = 344). The survival rates in *COLEC11-*high group were lower than that in *COLEC11-*low group at 10, 25, and 30 years with 33.5% versus 41.5%, 0% versus 13.1%, and 0% versus 5.4%, respectively, though the survival rate for *COLEC11-*high group at 20 years was reversed (Figure 8D). These results indicate that *COLEC11* gene expression is upregulated in melanomas and that high *COLEC11* expression has a trend toward poor survival.

Having demonstrated that CL-11 has a stimulatory effect on murine melanoma cell proliferation, we next sought to investigate whether CL-11 has stimulatory effects on human melanoma cell and other cancer cell proliferation. EdU proliferation assays were performed in human melanoma A375 cells, renal cell carcinoma Caki cells, and 3 liver cancer cell lines (MHCC-97H, SMMC-7721, HepG2) following the stimulation with human rCL-11. Treatment with rCL-11 significantly increased cell proliferation in all cell lines we examined (Figure 8, E–I), with the most striking effects on melanoma A375 cells (Figure 8, E and F) and liver cell lines MHCC-97H and SMMC-7721 (Figure 8, H and I). These results demonstrate that, as observed in mouse melanoma cells, CL-11 has a stimulatory effect on human melanoma cell and other cancer cell proliferation.

## Discussion

CL-11 has been shown to play important roles in homeostasis and pathogenesis of diseases and is involved in various biological processes; however, its role in tumor growth is currently unknown. In the present study, we demonstrate that CL-11 plays important roles in promoting melanoma cell proliferation and tumor growth. The protumor roles of CL-11 are supported by several findings of our study, these include: (a) melanoma growth is suppressed in *Colec11*^–/–^ mice; (b) CL-11 is required for melanoma cell proliferation, angiogenesis, and establishment of more immunosuppressive TME; and (c) CL-11 induces mitogenic kinase signaling and stimulates melanoma cell proliferation in vitro.

Emerging research has suggested that TME has a big impact on the tumor progression and efficacy of ICB therapy (39, 40). Malignant cells hijack key homeostasis functions to evolve in a surrounding environment to promote their survival and growth and avoid elimination by the immune system. The TME consists of multiple cell types (e.g., lymphocytes, myeloid lineage cells, cancer-associated fibroblasts), vasculature, and soluble factors (e.g., cytokines/chemokines, proteins with different biological activities), which are located in and around the tumor. Tumors are strongly interconnected with TME; tumor cells can influence the TME by releasing extracellular molecules, while immune cells in the TME can affect the tumor growth and metastasis. In general, TME exhibits immunosuppressive features, although it could be more or less immunosuppressive. The main features could include: (a) high proportion of myeloid lineage cells and low of lymphocytes, (b) lack of cytotoxic T cell infiltrating in the tumor core, and (c) production of more cytokines having tumor-promoting properties and fewer cytokines having antitumor properties. It has been shown that more immunosuppressive TME supports malignant development (41, 42) and is associated with tumor progression (43–45), while less immunosuppressive TME is associated with a favorable prognosis for the ICB therapy (40, 46). One striking finding we made in the present study is that CL-11 has a profound effect on the TME. Results from analyses of tumor-infiltrating leukocytes demonstrate that CL-11 deficiency led to a higher lymphocytes/myeloid cells ratio and a better cytotoxic T cell infiltration in the tumor core. Results from analyzing intratumor gene expression demonstrate that CL-11 deficiency led to upregulation of genes coding for several key cytokines/chemokines having antitumor properties ( *Ifng, Nos2, Il12, Ccl5, Cxcl1, Cxcl9*) and led to downregulation of the gene coding for protumor molecule (*Arg1*). Together, these findings strongly suggest that the CL-11 plays important roles in the establishment of immunosuppressive TME, contributing to tumor growth.

The observations that CL-11 has significant influence on the TME raise an important question of how CL-11 could regulate the TME. The answer is not yet clear. There are several mechanisms by which CL-11 could influence the TME. First, CL-11 may influence the TME through its antiinflammatory property in macrophages. In a separate study, we found that treatment of peritoneal macrophages with rCL-11 upregulated IL-10 production and downregulated LPS- or IFN-γ–induced proinflammatory cytokine (TNF-α, IL-12) production (unpublished data). Second, CL-11 may indirectly influence the TME through promotion of tumor cell proliferation and tumor growth. Third, CL-11 may influence the TME through regulation of antitumor immune responses. It has been shown that CL-11 deficiency caused more severe collagen-induced arthritis, which was associated with APC overactivation and enhanced Th1/Th17 responses (15). In addition, collectins (MBL, CL-11) has inhibitory effects on T cell proliferation and activation in CD3/CD28-activated T cells (47). Therefore, one could argue that CL-11 may play a role in antitumor immune responses. However, the observation that depleting CD8 T cells did not rescue the melanoma growth in *Colec11^–/–^* mice (Supplemental Figure 6) suggests that regulation of antitumor immune responses by CL-11 may not be the primary mechanism for CL-11–dependent tumor growth, further supporting the notion that CL-11–mediated tumor cell proliferation is the primary mechanism for CL-11–dependent tumor growth. Additionally, CL-11 influences antitumor immune responses, likely through local action. In the present study, we assessed the impact of CL-11 on systemic immune responses by analyzing leukocyte subsets in the spleen and peripheral blood of B16 melanoma-bearing WT and *Colec11^–/–^* mice. There was no significant difference in the proportions of CD4 T cell, CD8 T cell, and several myeloid lineage cells between the 2 groups of mice (Supplemental Figure 7). This observation aligns with the importance of resident T cells compared with circulating T cells in tumor immunity and immunotherapy (48).

TAMs play a central role in the TME and tumor progression. Once inflammatory monocytes are recruited to the tumor site, factors in the TME drive them to differentiate into TAMs that can be further reprogramed to M1- or M2-like phenotypes; TAMs with M2-like phenotype provide a microenvironment that favors tumor growth, tumor survival, and angiogenesis, whereas TAMs with M1-like phenotypes exert antitumor functions. TAMs can secrete numerous cytokines/chemokines, growth factors, and proangiogenic factors that influence the recruitment of leukocytes, tumor growth, angiogenesis, and tumor metastasis. In the present study, we explored the impact of CL-11 on TAMs’ phenotype and its potential influence on the TME by performing RNA-Seq analysis in isolated TAMs. TAMs derived from WT mice and *Colec11*-deficient mice exhibited a striking difference in their gene expression profiles, in the context of protumor growth factors, cytokines/chemokines, and M1/M2-related molecules. Upregulation of procancer genes (*Spp1, Igf1, Plgs2, Plau, Ctsk, Areg, Hbegf, Il1a, Ilb*) was found in TAM-WT compared with TAM-KO, and this aligns with the observation that CL-11 is required for tumor cell proliferation, tumor growth, and angiogenesis. A relatively high expression of chemokines responsible for myeloid cell recruitment and a relative low expression of chemokines for T cell and NK cell recruitment were found in TAM-WT, whereas an opposite pattern was found in TAM-KO. These findings align with the observations that higher proportion of myeloid lineage cells and lower proportion of lymphocytes (by flow cytometry) and lower gene expression of *Ccl5* and *Cxcl9* (by RT-PCR) within melanomas from WT mice compared with melanomas from *Colec11*^–/–^ mice. Furthermore, a relatively high expression of M1-related molecules and a relative low expression of M2-related molecules were found in TAM-WT, whereas an opposite pattern was found in TAM-KO, indicating CL-11–dependent reprograming of macrophages to the M2-like phenotype. Together, our findings in TAM RNA-Seq analysis strongly suggest that CL-11 has big impact on TAM phenotype and can potentially influence tumor growth, angiogenesis and the TME.

In addition to the effect on the TME, another striking finding we made in the present study is that CL-11 has a direct stimulatory effect on melanoma cell proliferation. Our findings that CL-11 binds to B16 cells —and that rCL-11 in melanoma cell cultures resulted in a significant increase in cell proliferation as well as ERK, JNK, and AKT signaling — strongly support the notion that CL-11 has stimulatory effects on tumor cell proliferation. To address the question of which receptor/molecule on melanoma is responsible for the action of CL-11, we investigated a hypothesis that CL-11 induced melanoma cell proliferation via ErbB receptors. Our findings that EGFR and HER3 are expressed in B16 melanoma cells (Supplemental Figure 5, A and B), and that rCL-11 in cell cultures rapidly results in phosphorylation of EGFR and HER3 — together with the fact that EGFR and HER3 are highly glycosylated proteins containing multiple N-glycans (49), which could be potential binding sites for CL-11 — provide strong evidence to support the notion that CL-11 could mediate melanoma cell proliferation through interaction with EGFR and HER3. Although HER3 alone may not mediate signaling, it may mediate signaling through heterodimerization with EGFR as previously suggested (50). It is therefore possible that CL-11 induces intracellular signal transduction through homodimerization of EGFR and heterodimerization of EGFR and HER3 to mediate melanoma cell proliferation.

**Figure 9:**
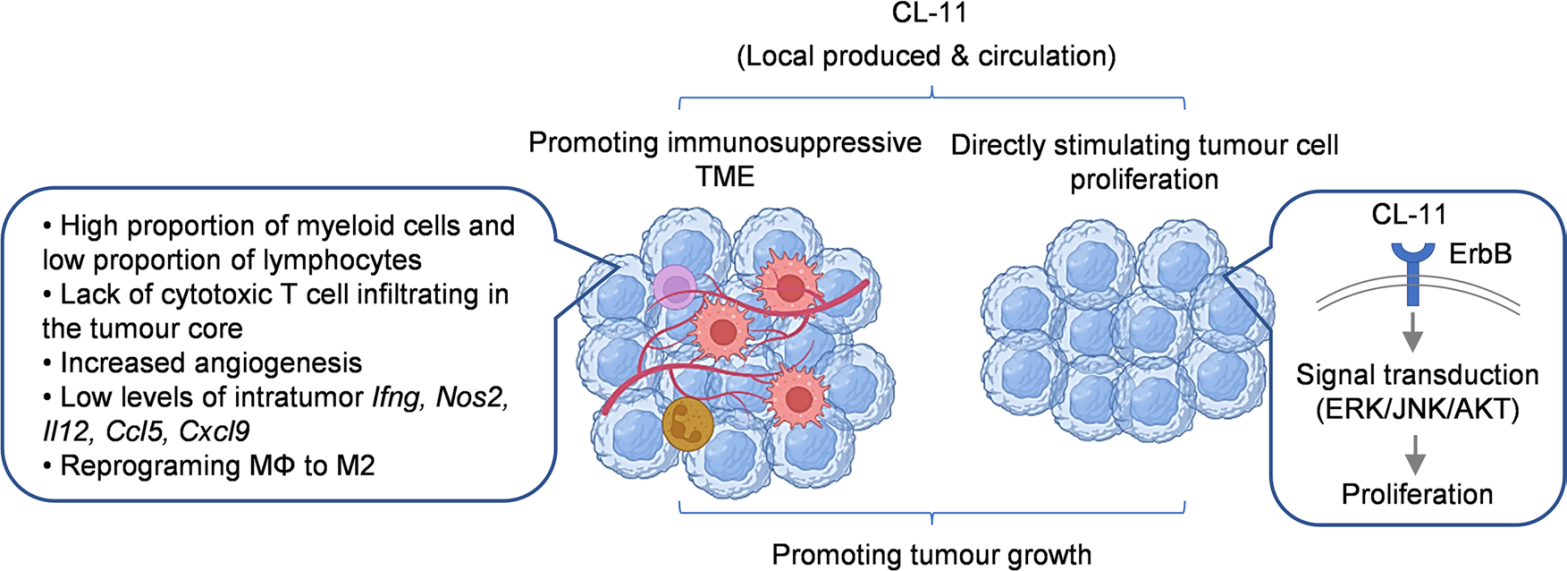
Proposed mechanism to describe the effects of CL-11 in promoting melanoma tumor growth. Based on our findings and literature interpretation, we propose that CL-11 in the tumor (locally synthesized and circulation derived) can contribute to melanoma tumor growth via 2 pathways: (a) CL-11–induced activation of mitogenic kinase signaling and stimulation of tumor cell proliferation, possibly through interaction with ErbB receptors (e.g., EGFR, HER2) on melanoma cells, and(b) CL-11–dependent promotion of immunosuppressive TME, characterized by a high proportion of myeloid cells and a low proportion of lymphocytes, lack of cytotoxic T cell infiltrating in the tumor core, increased angiogenesis, low levels of intratumor expression of cytokines/chemokines with antitumor properties (e.g., *Ifng, Nos2, Il12, Ccl5, Cxcl9*), and MΦ reprogramming to a M2 phenotype.

Based on our findings and literature interpretation, we propose a mechanism to describe the effects of CL-11 in promoting melanoma tumor growth (Figure 9). CL-11 in the tumor (locally synthesized and circulation-derived) can contribute to melanoma tumor growth via 2 main pathways: (a) CL-11–induced activation of mitogenic kinase signaling and stimulation of tumor cell proliferation and (b) CL-11–dependent promotion of immunosuppressive TME, characterized by high proportion of myeloid cells and low proportion of lymphocytes, lack of cytotoxic T cell infiltrating in the tumor core, increased angiogenesis, low levels of cytokines/chemokines having antitumor properties, and reprograming macrophages to M2.

In the present study, we evaluated the relevance of CL-11 in human melanomas. By analyzing *COLEC11* gene expression in human melanomas using multiple online data sets, we revealed that *COLEC11* gene expression is significantly upregulated in melanomas. It is unknown which types of cells in melanomas could be attributed to the upregulation of CL-11, as the data sets we analyzed include bulk RNA data. Survival analysis showed that high *COLEC11* expression has a trend toward poor survival, though there was no statistical significance over 30 years. It is still possible that the differential expression of the *COLEC11* gene among patients with melanoma alone does not dictate survival; instead, it is a factor that allows melanoma to originate and proliferate. We extended proliferation analysis in mouse melanoma cells to human melanoma cells. We found that CL-11 had stimulatory effects on the human melanoma cell proliferation, which is consistent with the observation in the mouse melanoma cells. In addition, ErbB receptors (EGFR, HER2, and HER3) were detected in A375 cells (Supplemental Figure 5, C and D), as they were in B16 cells. These observations suggest that the proposed mechanism of CL-11-stimulating cell proliferation in mouse melanoma cells could be operated in human melanoma cells. Therefore, although the observation of upregulation of *COLEC11* expression in human melanomas alone can be interpreted as “causative” or “consequence,” which — together with the findings that CL-11 has stimulatory effects in human melanoma cell proliferation, melanoma growth is suppressed in *Colec11^–/–^* mice, and high *COLEC11* expression has a trend toward poor survival — would support a pathogenic role for CL-11 in human melanoma. In the present study, we assessed whether the pathogenic role of CL-11 in melanomas is generalizable to other types of solid tumors. We performed proliferation assays in additional 4 human cancer cell lines, including a renal cancer cell line and 3 liver cancer cell lines, and found that CL-11 had striking effects on all the cancer cell lines (Figure 8, H–I). These findings, together with observations in mouse and human melanomas, strongly support the notion that CL-11 is a potent procancer factor in solid tumors.

In conclusion, our findings demonstrate a previously unknown role for CL-11 in promoting cancer cell proliferation and tumor growth, and they suggest that CL-11 acts mechanistically through 2 pathways, namely directly stimulating cancer cell proliferation and promoting establishment of more immunosuppressive TME, which has implications in human melanoma and other solid tumors. Targeting CL-11 has therapeutic potential in slowing or halting tumor growth.

## Methods

### Reagents.

The following antibodies were used in immunochemical staining: monoclonal rat anti–mouse CD45 (103120), CD11b (101202), and F4/80 (123102) (all from BioLegend); rat anti–mouse CD31(557355, BD Biosciences); rabbit anti-mouse CD3 (ab237721), CD8α (ab217344), VWF (ab6994), and rabbit anti–human COLEC11 (ab238585) (all from Abcam); rabbit anti–mouse H2-Ab1 (A18658, ABclonal); rat anti–mouse CD68 (FA11) (Bio-Rad); goat anti–mouse CD206(AF2535) (from R&D systems); rabbit anti–mouse Ki67 (9129, Cell Signaling Technology); Alexa Fluor 488 goat anti–rat IgG (catalog 405418), Alexa Fluor 555 goat anti–rat IgG (catalog 405420), and Alexa Fluor 647 donkey anti–rabbit IgG (catalog 406414) (all from BioLegend); and Alexa Fluor 488 goat anti–rabbit IgG (catalog 4412) and Alexa Flour 594 goat anti–rabbit IgG (catalog 8889) (both from Cell Signaling Technology). The following fluorochrome-conjugated monoclonal antibodies were used for the flow cytometry detection: FcR-blocking antibody (CD16/32, 2.4G2, BD Biosciences), FITC-conjugated anti-mouse CD3ε (catalog 100306), CD11b (catalog 101206), PE-conjugated rat anti–mouse CD8a (catalog 100708), CD4 (catalog 100407), CD19 (catalog 115508), Ly6G (catalog 127608), F4/80 (catalog 123110), PE/Cy7-conjugated anti–mouse Ly-6C Ab (catalog 128018), APC-conjugated rat anti–mouse NK-1.1 (catalog 108710), CD45 (catalog 103112), and CD25 (catalog 102012) (all from BioLegend). The following antibodies were used in signaling pathway studies: anti–phospho-ERK1/2 (Thr202/Tyr204, 197G2), anti–phospho-Akt (Ser473, D9E), anti–phospho-JNK (Thr183/Tyr185, 81E11), anti–phospho-HER1 (Tyr1068, D7A5), anti–phospho-HER2 (Tyr1221/1222, 6B12), anti–phospho-HER3 (Tyr1289, D1B5), and anti-ERK1/2, anti-Akt, anti-JNK, anti-HER1(D38B1), anti-HER2 (D8F12), anti-HER3 (D22C5), and anti-HER4 (111B2) (all from Cell Signaling Technology). Ultra-LEAF Purified anti–mouse CD8a Antibody (100763, BioLegend) was used in CD8 depletion experiments. We also used the following reagents: cell culture medium, FCS, TRIzol, Fast SYBR Green Master Mix, M-PER mammalian protein extraction reagent, BCA protein assay kit, Alexa Fluor 488 Click-iT EdU Cell Proliferation Kit (catalog C10337) and Alexa Fluor 555 Tyramide SuperBoost Kit (catalog B40933) (all from Thermo Fisher Scientific); recombinant human and murine CL-11 (endotoxin ≤ 1 EU per 1 μg of protein; Bon Opus Biosciences); BSA (endotoxin < 10 EU per 1 μg of protein), L-fucose (catalog F2262) (both from Sigma-Aldrich); and D-luciferin (R&D system). Cancer lines (B16, YUMM1.7, A375, Caki, MHCC-97H, SMMC-7721, HepG2) were from the American Type Culture Collection (ATCC).

### Mice.

Homozygous *Colec11^–/–^* mice on C57BL/6 background (25) were obtained from Mutant Mouse Resource and Research Centers (UCD, Davis, California, USA) and back-crossed onto C57BL/6 for at least 10 generations. WT littermates were used as controls. Male mice (8–10 weeks of age) were used in all experiments unless specified otherwise. All mice were maintained in specific pathogen–free conditions. Immune profiling revealed that there is no significant difference in basal levels of T cell and B cell activities and in serum cytokines between naive *Colec11^−/−^* mice and WT mice (15). The Ethics Review Committee for Animal Experimentation at Xi’an Jiaotong University approved and oversaw all mouse experiments.

### Murine s.c. tumor models.

Murine melanoma cell line B16 was cultured in DMEM with 10% FCS. Murine melanoma cell line YUMM1.7, which was derived from the *Braf^V600E^Pten*^–/–^*Cdkn2a*^–/–^ mouse model (26) that resembles human melanoma, was cultured in DMEM/F-12 with 10% FCS and supplemented with 1% nonessential amino acids. The cells were harvested and washed 3 times with PBS and resuspended in PBS. Mice were injected s.c. on 1 flank with B16 cells or YUMM1.7 cells (2.5 ***×*** 10^5^ cells in 100μL PBS/mouse). Tumor sizes were measured by callipers daily from day 6, and tumor volume (V) was calculated using the formula: V (mm^3^) = L ***×*** W^2^ ***×*** 0.5, in which L corresponds to the largest diameter (length) and W to the smallest diameter (width) of the tumor in mm, respectively. Mice were sacrificed on day 14, and tumors and spleen were excised. Tumor size and weight, tumor cell proliferation, angiogenesis, composition of tumor-infiltrating leukocytes, and intratumor gene expression were assessed. In L-fucose treatment experiment, mice were treated with L-fucose (1 g/kg) or saline daily by i.p. injection starting 24 hours after the injection of B16 melanoma cells. Single-cell suspensions of melanomas were prepared by mechanical disruption with 70 mm nylon mesh. Single-cell suspensions were used for flow cytometry and isolation of TAMs by flow sorting.

### Tumor monitoring by in vivo bioluminescence.

Mice were inoculated with luciferase-labeled B16 melanoma cells (B16-luc cells, 2.5 ***×*** 10^5^ in 100 μL PBS, s.c.) on day 0. On day 14, mice were shaved, anesthetized, and administered with 30 μg D-luciferin by i.p. injection. Bioluminescence images were acquired using the IVIS Lumina XRMS Series III (Xenogen). All images were analyzed using Living Image software (Xenogen). Average radiance (p/s/cm^2^/sr) is used to determine the tumor burden, which refers to the number of photons (p) per second that are leaving a square centimeter of tissue and radiating into a solid angle of 1 sr.

### Flow cytometric analysis.

Single-cell suspensions of tumor or spleen were prepared from melanoma-bearing mice (on d14). Cells were preincubated with FcR blocking antibody (CD16/32) and incubated with rat anti–mouse APC-conjugated CD45, NK1.1, FITC-conjugated CD3ε, CD11b, PE-conjugated Ly6G, CD8α, CD4, CD19, and PEcy7-conjugated Ly6C antibodies or the appropriate isotype control antibodies at 4°C for 20 minutes. All flow cytometric analysis was performed using Calibur Flow Cytometer (BD Biosciences) and FlowJo software.

### IHC.

OCT-embedded frozen tumor sections (4 μm) were immersed in PBS for 5 minutes and then blocked with 10% goat serum for 30 minutes at room temperature. Sections were incubated with primary antibody (e.g., rabbit anti–mouse Ki67, VWF, CD3, CD8α, and H2-Ab1; goat anti–mouse CD206; rat anti–mouse CD45, CD31, CD11b, F4/80, CD68; and rabbit anti–human COLEC11, which can cross-react with mouse Colec11) or isotype control antibody overnight at 4°C and were then incubated then with secondary antibody (e.g., Alexa Fluor 488 goat anti-rat, Alexa Fluor 555 goat anti-rat, Alexa Fluor 488 goat anti-rabbit, Alexa Flour 594 goat anti-rabbit) for 1 hour at room temperature. Nuclei were stained with DAPI. The stained sections were digitally captured and examined by a Leica confocal microscope (Leica SP8, Germany). OCT-embedded frozen tumor sections (4 μm) were immersed in PBS for 5 minutes and then blocked with 10% goat serum for 30 minutes at room temperature. Sections were incubated with primary antibody (i.e., rabbit anti–mouse Ki67, VWF, CD3, CD8α; rat anti–mouse CD45, CD31, CD11b, F4/80; and rabbit anti–human COLEC11) or isotype control antibody overnight at 4°C and then with secondary antibody (i.e., Alexa Fluor 488 goat anti-rat, Alexa Fluor 555 goat anti-rat, Alexa Fluor 488 goat anti-rabbit, Alexa Flour 594 goat anti-rabbit) for 1 hour at room temperature. Nuclei were stained with DAPI. The stained sections were digitally captured and examined by a Leica confocal microscope (Leica SP8). The Ki67^+^ cells and positively stained VWF and CD31 areas were calculated using ImageJ software (NIH), at 200***×*** magnification. The CD206^+^ or H2-Ab1^+^ cells, and fluorescence intensity of CD206 or H2-Ab1 in CD68^+^ cells was calculated using ImageJ software at 200***×*** magnification. Intensity index values of CD206 and H2-Ab1 were calculated by dividing the total fluorescence intensity of CD206 or H2-Ab1 in CD68^+^ cells by the number of total CD68^+^ cells in the given area (0.34 mm^2^) and were then normalized by setting the mean value in WT group as 1. Image capture and analysis were conducted by 2 researchers in a blinded fashion.

### RNA isolation, RT-PCR, and qPCR.

Total RNA was extracted from melanomas using TRIzol reagent. Complementary DNA (cDNA) was synthesized in a 25 μL reverse-transcription reaction mix consisting of 2 μg of total RNA, 0.5 μg Oligo(dT)_12–18_ primer, 10 mM nucleotide mixture, 25 U recombinant RNasin ribonuclease inhibitor, and 200 U M-MLV reverse transcriptase. Conventional PCR was carried out in a 25 μL volume with cDNA reflecting 0.15 μg RNA, 12.5 μL GoTaq G2 Green master mix, and 2 μM of each primer. DNA was denatured at 94°C, annealed at 60°C, and extended at 72°C, with a total of 40 reaction cycles. Amplified PCR products were electrophoresed in a 1.4% agarose gel and photographed under ultraviolet light. qPCR was performed with a DyNAmo HS SYBR Green qPCR kit and an MJ Research PTC-200 Peltier Thermal Cycler (Bio-Rad) according to the manufacturers’ instructions. Each sample was amplified in duplicate. The relative gene expression was analyzed using the 2^–ΔΔCt^ method. The control sample was a single tumor tissue from WT mice. The test samples were individual tumor tissues from both WT and *Colec11^–/–^* mice. The information for primer sequence is given in Supplemental Tables 1 and 2.

### Detection of CL-11 binding to B16 cells.

For immunochemical staining, the Tyramide SuperBoost system with streptavidin was used. B16 were seeded on coverslips in 24-well plate at a density of 1.5 ***×*** 10^5^/well and cultured for 24 hours. The cells were fixed with 4% paraformaldehyde (PFA) for 15 minutes at room temperature (but not permeabilized). The cells were then incubated in serum-free culture medium supplemented with 2% BSA alone or containing biotin rCL-11 (1,200 ng/mL) at 4°C overnight, followed by incubated with HRP-conjugated streptavidin for 1 hour at room temperature and Alexa Fluor 555 Tyramide reagent detection according to the manufacture’s instruction. The staining was examined by confocal microscope (Leica SP8). For flow cytometry, single-cell suspensions of B16 cells were incubated with 1,200 ng/mL of rCL-11 at 37°C for 1 hour. After thoroughly washing, the cells were fixed with 4% PFA for 15 minutes. The cells were then preincubated with FcR blocking antibody (CD16/32) and incubated with rabbit anti–human CL-11 antibody at 4°C for 1 hour, followed by the secondary Alexa Fluor 647 donkey anti-rabbit antibody. The stained cells were analyzed using Calibur Flow Cytometer. In some experiment, rCL-11 was incubated with L-fucose (2 mg/mL) for 30 minutes at room temperature and then applied in the assays of binding CL-11 to B16 cells as described in the above section.

### Proliferation assay.

B16, A375, or Caki cells were seeded in coverslips of a 24-well plate at a density of 2 × 10^4^/well and cultured in DMEM with 10% FCS for 24 hours. The cells were further cultured in the DMEM with 5% FCS in the presence of rCL-11 or control (BSA) for 48 hours. At the end of rCL-11 treatment, cells were incubated with serum-free DMEM containing 30 μM EdU for 1 hour and fixed in 4% PFA for 15 minutes. Incorporated EdU was detected by a Click-iT EdU Alexa Fluor 488 Imaging kit according to manufacturer’s instructions (Thermo Fisher Scientific, C10337). Pictures were taken by a Leica SP8 confocal microscope. The DAPI^+^ nuclear (representing the total cell number) and EdU^+^ nuclear (representing the proliferating cell number) were counted using ImageJ software. The proliferation rate was calculated using the following formula: proliferation rate = (EdU^+^ nuclear number/DAPI^+^ nuclear number) ***×*** 100%.

The human HCC cell lines HepG2, SMMC7721, and MHCC97H were seeded in a black 96-well plate with transparent flat bottom (3904, Corning) at a density of 6 ***×*** 10^3^/well and cultured in DMEM with 5% FCS in the presence of rCL-11 or control (BSA) for 40 hours. Cells were incubated with serum-free DMEM containing 50 μM EdU for 8 hours and fixed in 4% PFA for 15 minutes. Incorporated EdU was detected by a Click-iT EdU Alexa Fluor 488 Imaging kit according to manufacturer’s instructions. The fluorescence intensity was measured using a fluorescence plate reader (SpectraMax i3, Molecular Devices) with excitation at ~480 nm and emission at ~520 nm. The fluorescence value of the reagent blank (EdU^+^ Click-iT reaction buffer only) was subtracted from that of each sample.

### Western blotting.

B16 cells were seeded in a 6-well plate at a density of 2 × 10^6^ /well and cultured in the full medium for 24 hours. After serum starvation for 1.5 hours, B16 cells were incubated with rCL-11 or BSA at different time points and lysed using M-PER mammalian protein extraction reagent containing proteinase and phosphatase inhibitor cocktail mixture on ice. Supernatants of cell lysates were collected after centrifugation at 14,000*g* at 4°C for 15 minutes. Protein concentrations were determined by BCA protein assay kit according to the manufacturer’s instruction. Equal amounts of protein (30 μg per lane) were subjected to SDS-PAGE electrophoresis. After separation, the proteins were transferred from the gel on to PVDF membrane. The membranes were incubated with primary antibodies overnight at 4°C, followed by incubation with HRP-conjugated secondary antibody for 1 hour. Protein bands were detected using Amersham ECL Select detection reagent. Quantification of protein bands on the gel was performed by measuring the intensity of individual band using ImageJ software. The relative amount of phosphorylated protein was generated by normalization to the total protein of respective molecules.

### Sorting of TAMs.

Single-cell suspensions of tumors were resuspended in PBS containing 1% BSA and blocked with anti-CD16/CD32; they were then stained with APC-conjugated CD45 and PE-conjugated F4/80 and then sorted by BD FACS Aria II. The purity of the sorted CD45^+^F4/80^+^ cells was consistently more than 95%, as determined by flow cytometry.

### RNA-Seq analysis.

Total RNA extraction, cDNA library construction, and sequencing were performed by the Beijing Genomics Institute (Shenzhen, China) using BGISEQ-500 platform. High-quality reads were aligned to the mouse reference genome using Bowtie2. Expression levels for each of the genes were normalized to fragments per kilobase of exon model per million mapped reads (FPKM) using RNA-Seq by Expectation Maximization (RSEM). The assembled genes were annotated in Kyoto Encyclopedia of Genes and Genomes (KEGG; https://www.kegg.jp). Further analysis of annotated genes was conducted in BGI Dr. Tom platform (https://biosys.bgi.com). A combination of fold change ≥ 2 and *q* ≤ 0.05 was used to define DEGs. The heatmap was drawn by Heatmapper (51) using (fold change ≥ 1.5) DEGs in different samples. The transcriptomic data discussed in this study have been deposited in NCBI’S Gene Expression Omnibus (GEO) and are accessible through GEO series accession no. GSE193454.

### Statistics.

Data are shown as mean ± SEM, unless otherwise specified. Unpaired 2-tailed *t* test was used to compare the means of 2 groups. Paired *t* test was used to compare the means of matched pairs. One-way or 2-way ANOVA was used to compare the means of more than 2 independent groups. Patient survival was analyzed using stratified multivariate log-rank test. All the analyses were performed using GraphPad Prism 9 software, unless otherwise specified. *P* < 0.05 was considered to be significant.

### Study approval.

Animal experiments were performed according to protocols approved by the school Ethics Review Committee for Animal Experimentation in Xi’an Jiaotong University (no. 2017-411).

## Author contributions

JXW, BC, NM, and KYW conducted experiments, acquired data, analyzed data, and wrote the manuscript. WW, XD, WBC, CFL, YFG, TYD, XYM, and QY conducted experiments. ZFL contributed data interpretation, analysed data, and provided critical reading of the manuscript. WZ designed research studies and wrote the manuscript. KL designed research studies, analysed data, and wrote the manuscript. The co–first authorship order was decided considering the study is JXW’s PhD project and by flipping a coin for BC and NM.

## Supplementary Material

Supplemental data

## Version 1 03/08/2023

Electronic publication
