# Supplementary material for: Collectin-11 promotes cancer cell proliferation and tumor growth
Source: JCI Insight. 2023 Mar 8;8(5):e159452. doi: 10.1172/jci.insight.159452 (PMC10077485; doi:10.1172/jci.insight.159452)
Supplement: Supplemental data [file jciinsight-8-159452-s210.pdf]

sFigure 1. Detection of CL-11 in melanoma tumours

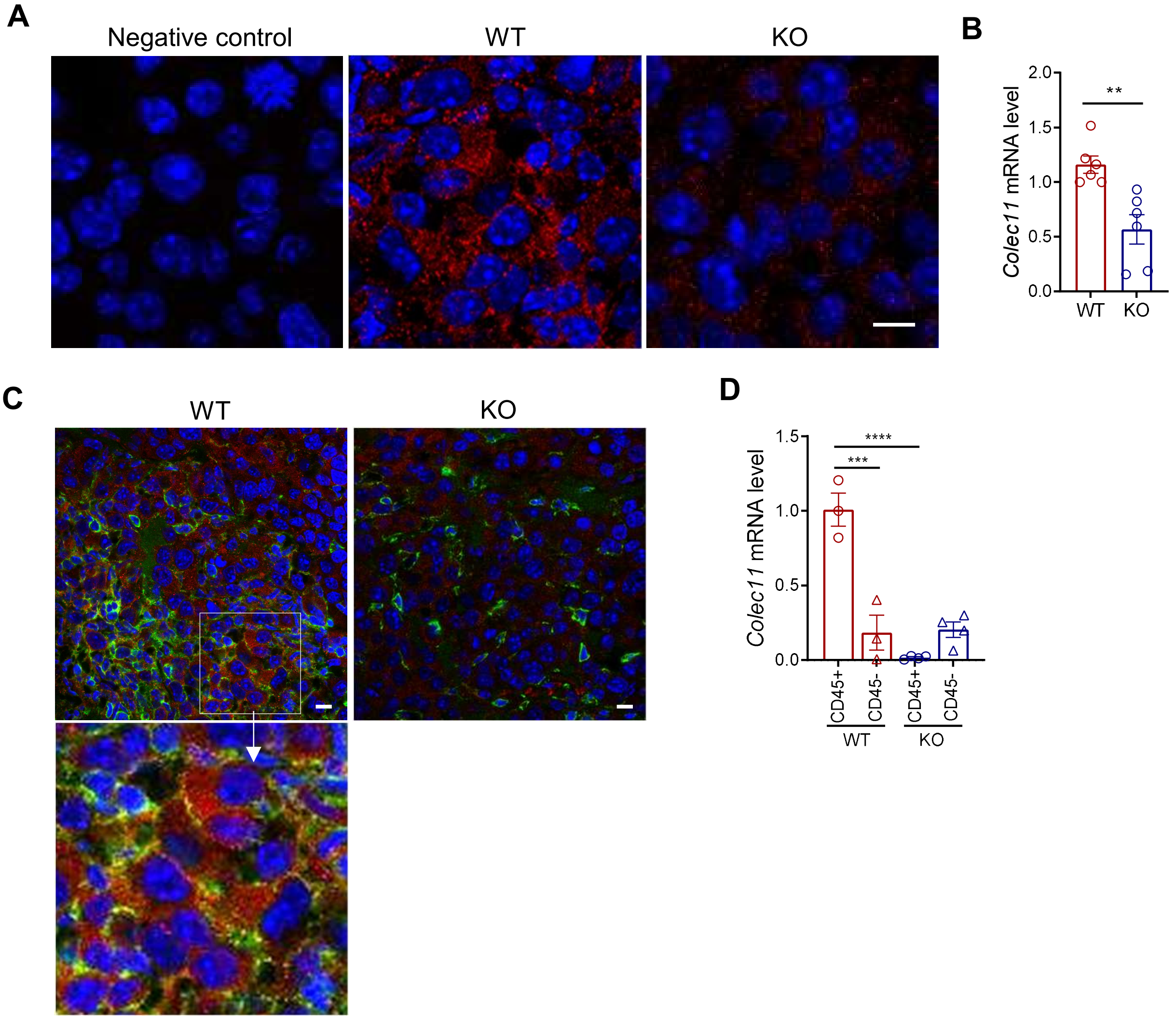

Tumours excised from *Colec11*$^{+/+}$ (WT) or *Colec11*$^{-/-}$ (KO) mice (d14) were used for analysis of CL-11 expression. (**A**) Representative microscopy images of immunochemical staining of CL-11 (red)/DAPI (blue) (n=4). Negative control: the staining was performed in 2nd antibody alone. Scale bar: 10 μm. (**B**) RT-qPCR analysis for CL-11 mRNA in tumour tissues. Data were analyzed by Unpaired t-test (n= 6 mice/group). (**C**) Representative microscopy images of immunochemical co-staining of CL-11 (red)/CD45 (green) and DAPI (blue) (n=4). Top panel: lower magnification images. Scale bars: 10 μm. Lower panel: a higher magnification image corresponding to the boxed region in the above image. (**D**) RT-qPCR analysis for CL-11 mRNA in isolated CD45$^+$ cells and CD45$^-$ cells from tumour tissues. Data are expressed as Mean±SD and were analyzed by Data were analyzed by One-way ANOVA with Tukey's multiple comparisons test (n= 3 or 4 mice/group). **, P<0.01; ****, P<0.0001.

sFigure 2. The proportions of CD19$^+$ B cells and CD4$^+$CD25$^+$ cells in TME

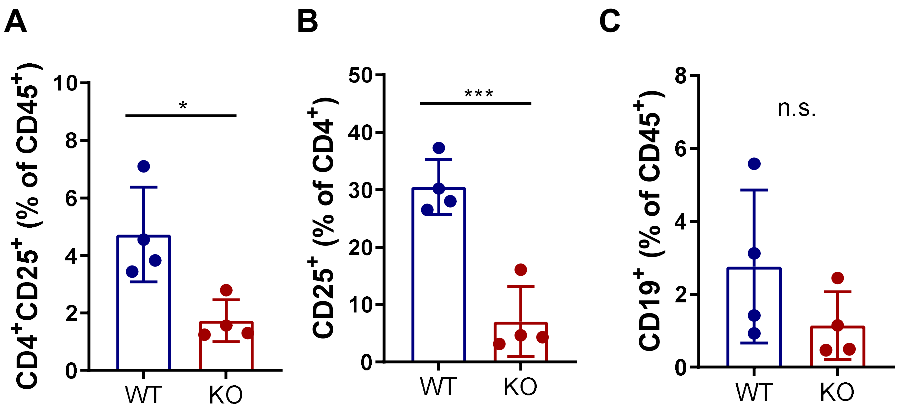

Tumours excised from *Colec11*$^{+/+}$ (WT) or *Colec11*$^{-/-}$ (KO) mice (d14) were used for analysing the population of CD19+ B cells and CD4+CD25+ Treg cells by flow cytometry. (**A**) The proportion of CD4+CD25+ cells in CD45+ cells. (**B**) The proportion of CD25+ cells in CD4+ T cell compartment. (**C**) the proportion of CD19+B cells in CD45+ cells. Data are expressed as Mean±SD and were analyzed by Unpaired t-test (n= 4 mice per group). Each dot represents an individual mouse. *, P<0.05; ***, P<0.001.

sFigure 3. *Colec11$^{-/-}$* mice exhibit more more CD3^{+} infiltrates and much less CD11b^{+} infiltrates in YUMM1.7 tumour

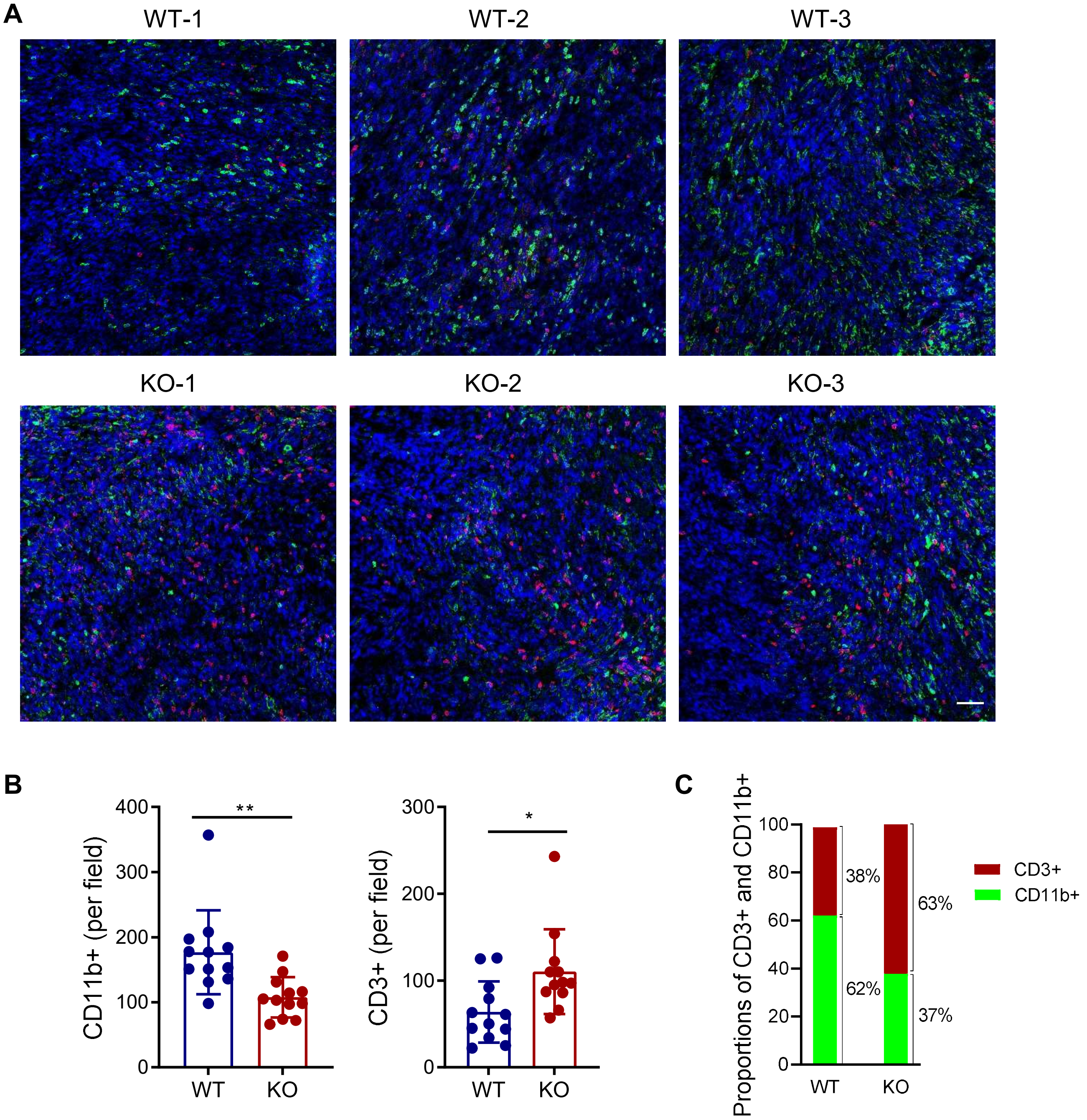

YUMM1.7 tumours excised from *Colec11$^{+/+}$* (WT) or *Colec11$^{-/-}$* (KO) mice (d14) were used for analysing leukocyte infiltrates by immunochemical staining. (**A**) Representative microscopy images of staining for CD11b (green)/CD3 (red)/DAPI (blue) (3 mice per group). Scale bar: 50 μm. (**B**) Quantification of CD11b+ and CD3+ cells corresponding to the groups in A. Data are expressed as number of CD11b+ or CD3+ cells per field (0.34 mm2). Data were analyzed by Unpaired t-test (n=12, 4 fields each mouse, 3 mice per group). (**C**) A bar chat representing proportion of CD11b+ and CD3+ shown in **A** and **B.** *, P<0.05; **, P <0.01.

sFigure 4. RNA-seq analysis in TAMs from WT and *Colec11*$^{-/-}$ mice

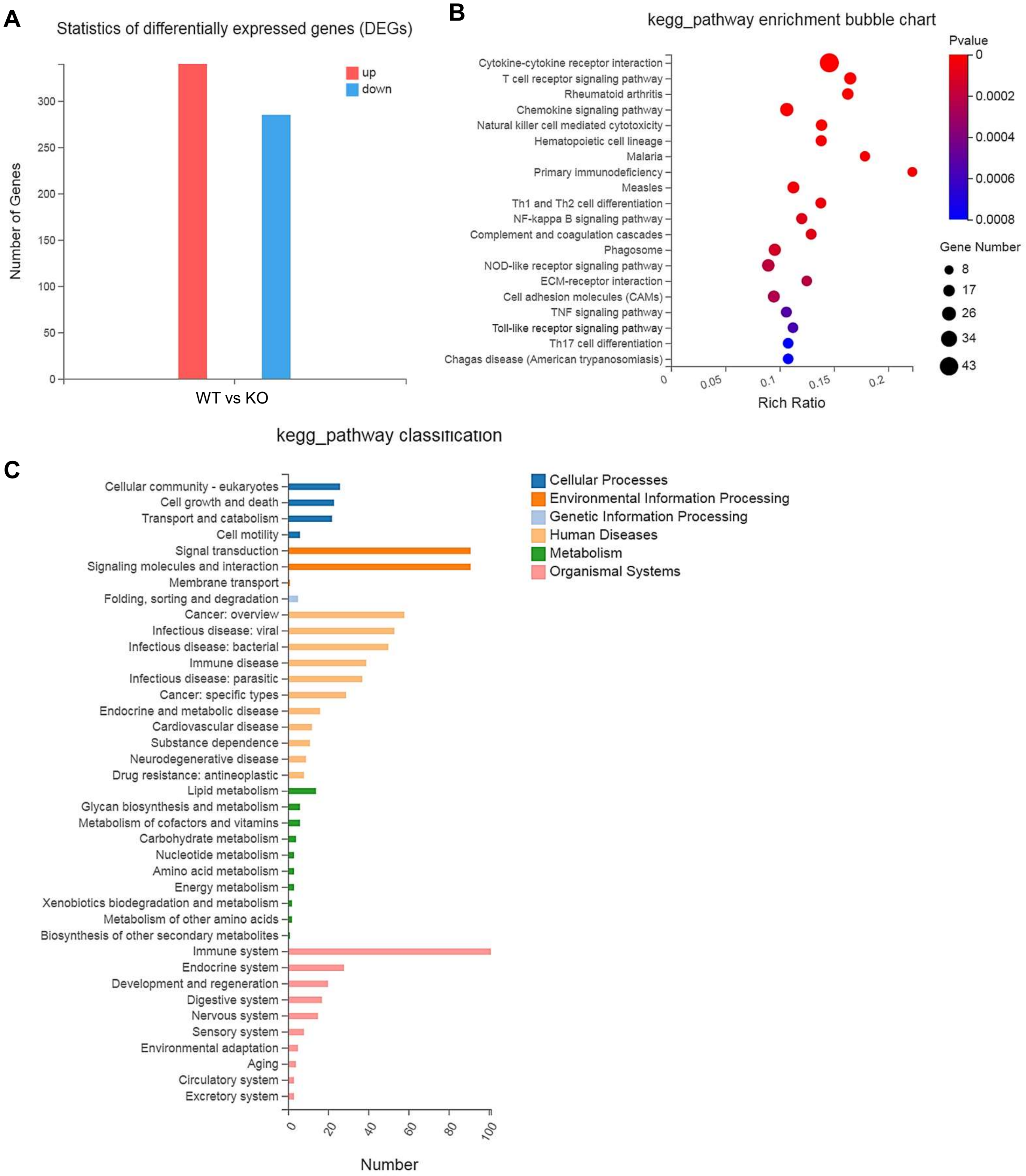

RNA-Seq analysis identified 625 differentially expressed genes (DEGs) (Log2FC≥1) between *Colec11*$^{+/+}$ (WT) and *Colec11*$^{-/-}$ (KO) mice. These DEGs are mainly classified into "Immune system", "Signal transduction" "Signalling molecules and interaction" and "Cancer" pathways. (**A**) Number of DEGs. (**B**) KEGG pathway enrichment analysis of DEGs. (**C**) KEGG pathway classification analysis of DEGs.

sFigure 5. Detection of ErbB receptors in murine and human melanoma cells

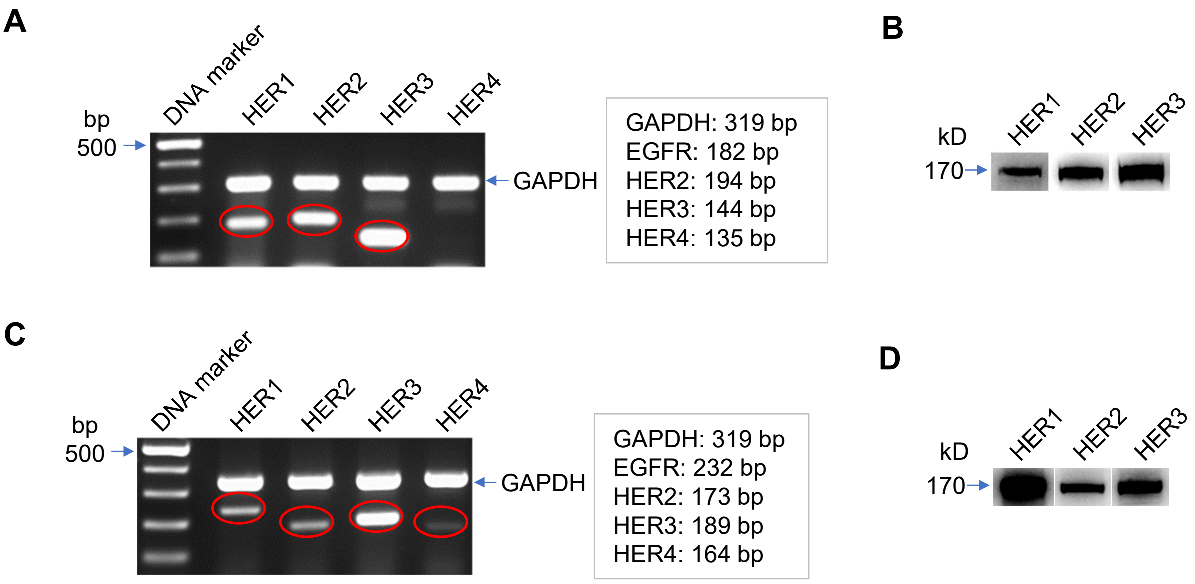

(**A, B**) Detection of ErbB receptors in cultured murine B16 melanoma cells. (**A**) RT-PCR. (**b**) Western blot. HER1, HER2 and HER3, but not HER4 were positively detected. (**C, D**) Detection of ErbB receptors in cultured human (A375) melanoma cells. (**C**) RT-PCR. (**D**) Western blot. HER1, HER2 and HER3 were positively detected. A weak expression of HER4 was also detected by RT-PCR. GAPDH was used as an internal control in PCR.

sFigure 6. Effects of depleting CD8 T cells on melanoma growth in WT and *Colec11$^{-/-}$* mice

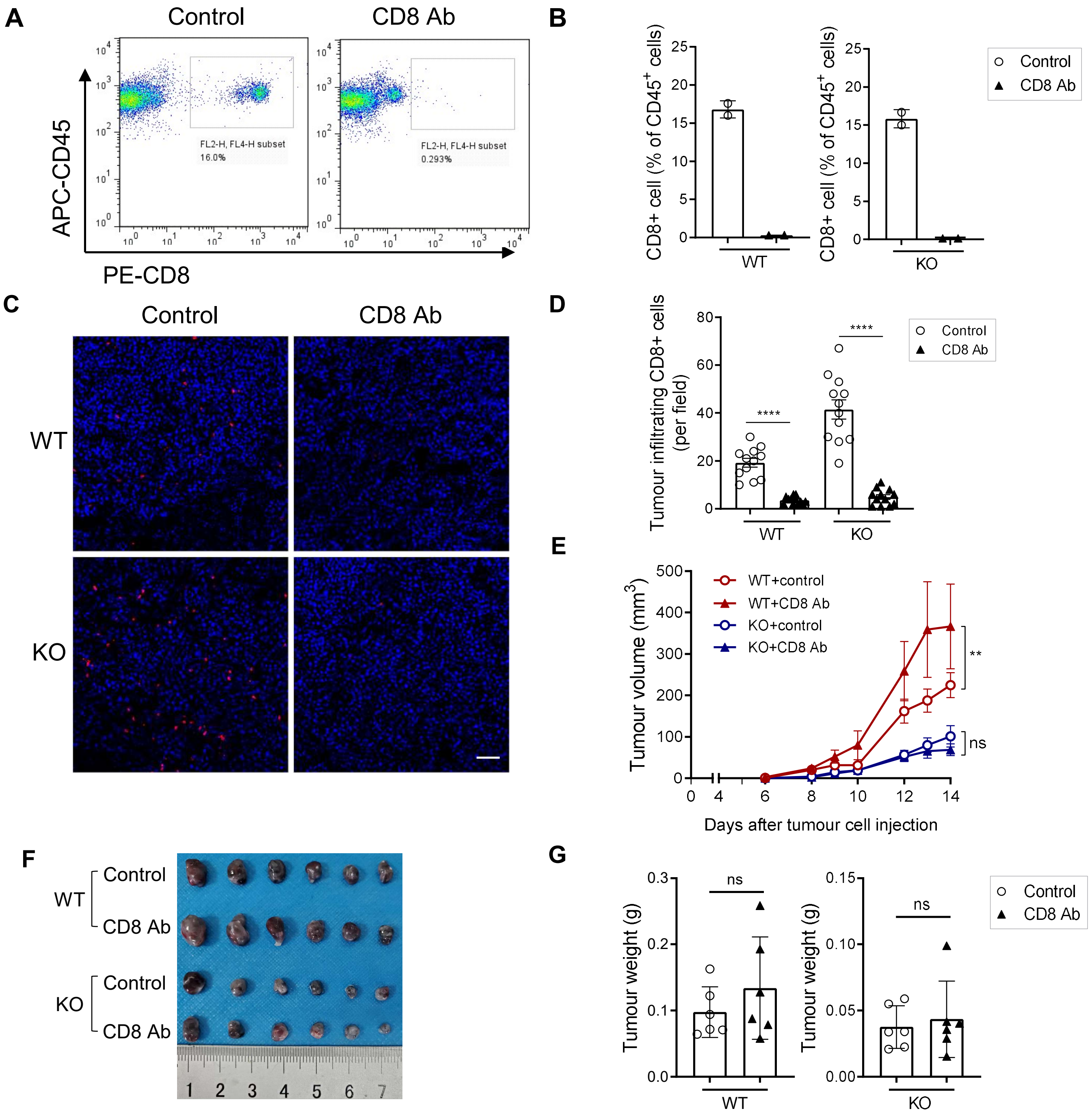

(**A, B**) Verifying the efficacy of CD8 T cell depletion in normal mice. Flow cytometry analysis of CD8+ T cells in peripheral blood of normal *Colec11$^{+/+}$* (WT) or *Colec11$^{-/-}$* (KO) mice 24h after i.p injection of anti-murine CD8 antibody (CD8 Ab)(100 mg/mouse) or control (saline). (**A**) Representative dot plot graphs in control and CD8 Ab treated mice. (**B**) Quantification of CD8+ T cells in CD45+ compartment in control or CD8 Ab treated WT and KO mice (2 mice/group). (**C-G**) B16 melanoma growth in WT and KO mice treated with CD8 depleting antibody or control (saline) (by i.p injection, starting 48 h before and 0, 3, 6, 9, 12 days after the inoculation of B16 cells). (**C, D**) Tumour infiltrating CD8 T cells. (**C**) Representative images of immunochemical staining for CD8 (red)/DAPI (blue). Scale bar: 50 μm. (**D**) Quantification of CD8+T cells. Data were analyzed by two-way ANOVA with multiple comparisons test (n=12, 3 mice, 4 field from each tumor/each mouse. (**E**) Tumour volume (d6 to d14). Data were analyzed by two-way ANOVA with multiple comparisons test. Each symbol represents the mean of a group of mice (n=6 mice/group) (**F**) images of tumours (d14) (**G**) Tumour weight (d14). Data were analyzed by Unpaired t test test (n=6 mice/group). **, P<0.01; **** P<0.0001.

sFigure 7. Analysis of systemic leukocytes subpopulations from B16 melanoma-bearing mice

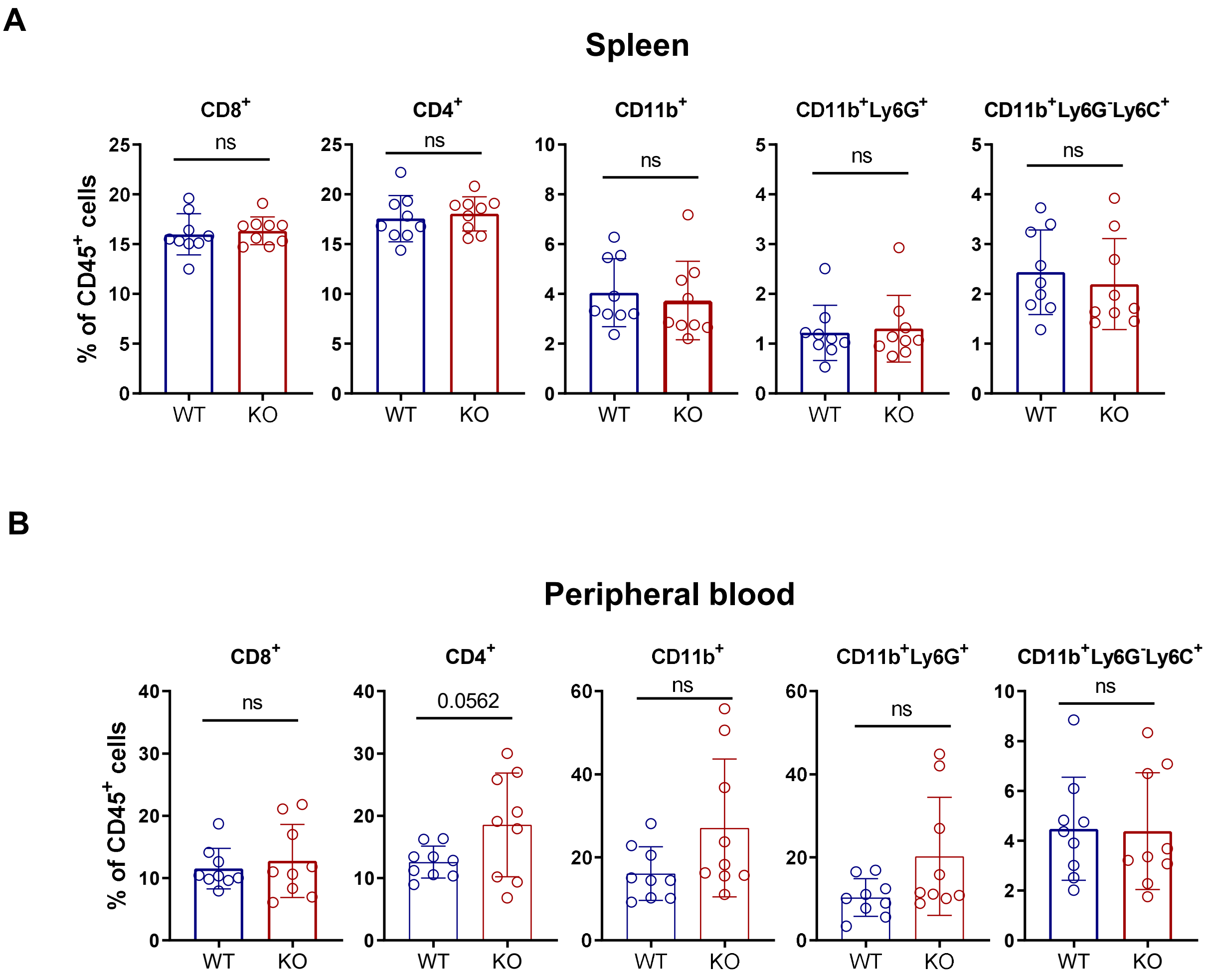

Flow cytometry analysis for subsets of leukocytes in spleen (**A**) and peripheral blood (**B**) from B16 melanoma-bearing *Colec11*$^{+/+}$ (WT) or *Colec11*$^{-/-}$ (KO) mice (on d14), showing major leukocyte populations are comparable between the KO and WT mice. All data are expressed as Mean ± SD and were analyzed by unpaired t-test (n= 9 mice per group). Each dot represents an individual mouse.

Supplemental Table 1

PCR primer sequences and product sizes (mouse)

**Gene id**	**Oligonucleotide Sequence (5' → 3')**	**Product Size (bp)**	**Gene bank code**
IFNG	ACAGCAAGGCGAAAAAGGATG<br>AATCTCTTCCCCACCCCGA	175	NM_008337.4
NOS2	GGGCAGTGGAGAGATTTTGC<br>CTCCAGAGGGGTAGGCTTGT	142	NM_001313922.1
IL12A	CCAGGTGTCTTAGCCAGTCC<br>GGTTTGGTCCCGTGTGATGT	134	NM_008351.3
ARG1	TTGTGAAGAACCCACGGTCT<br>AGATGCTTCCAACTGCCAGA	129	NM_007482.3
CCL5	TGCTCCAATCTTGCAGTCGT<br>GCAAGCAATGACAGGGAAGC	160	NM_013653.3
CXCL9	CGAGGCACGATCCACTACAA<br>GAGTCCGGATCTAGGCAGGT	125	NM_008599.4
CX3CL1	CTACTAGGAGCTGCGACACG<br>AAGCCACTGGGATTCGTGAG	198	NM_009142.3
COLEC11	TGGAAAAATTGGTCCCATTGGC<br>CATCTCCCCAATAGCCTTCCTC	130	NM_001313978.1
ACTB	CACACCCGCCACCAGTTCG<br>ACATGCCGGAGCCGTTGTC	70	NM_007393.5
GAPDH	GAGCGAGACCCCACTAACAT<br>GTGGCAGTGATGGCATGGAC	317	NM_001289726.1
EGFR	GAGTGACTGTCTGGTCTGCC<br>AGCCATGATCTGTCACCACG	182	NM_207655.2
ERBB2	CATCGTGAGAGGGACTCAGC<br>GGTAGCAGAGCTGAGGGTTC	194	NM_001003817.1
ERBB3	TGCTTACGGGACACAATGCT<br>TCCCATCGTAGACCTGGGTT	144	NM_010153.2
ERBB4	CTCTCTGACCTGGAACAGCAG<br>GACTTCTCGGATAGACCGCAG	135	NM_010154.2

Primer-1 is identical to the coding strand; primer-2 is complementary to the coding strand. All primer pairs sequences contain intron(s).

Supplemental Table 2

PCR primer sequences and product sizes (human)

Gene id	Oligonucleotide Sequence (5' → 3')	Product Size (bp)	Gene bank code
GAPDH	GAGTCCACTGGCGTCTTCAC	339	NM_002046.7
	GGCAGGGATGATGTTCTGGA		
EGFR	GACAGGCCACCTCGTCG	232	NM_005228.5
	TCGTGCCTTGGCAAACTTTC		
ERBB2	AGAGGACGAGTGTGTGGGC	173	NM_004448.4
	TGGCATTCACATACTCCCTGG		
ERBB3	CTTGCCTCGATGTCCTAGCC	189	NM_001982.4
	GGTCACACTCAGGCCATTCA		
ERBB4	GTTCAGGATGTGGACGTTGC	164	NM_005235.3
	GCCTCCAGCACATTCTCGAT		

Primer-1 is identical to the coding strand; primer-2 is complementary to the coding strand. All primer pairs sequences contain intron(s).
